# *ABCA7* p.G215S as potential protective factor for Alzheimer's disease

**DOI:** 10.1016/j.neurobiolaging.2016.04.004

**Published:** 2016-10

**Authors:** Celeste Sassi, Michael A. Nalls, Perry G. Ridge, Jesse R. Gibbs, Jinhui Ding, Michelle K. Lupton, Claire Troakes, Katie Lunnon, Safa Al-Sarraj, Kristelle S. Brown, Christopher Medway, Naomi Clement, Jenny Lord, James Turton, Jose Bras, Maria R. Almeida, ARUK Consortium, Peter Passmore, David Craig, Janet Johnston, Bernadette McGuinness, Stephen Todd, Reinhard Heun, Heike Kölsch, Patrick G. Kehoe, Emma R.L.C. Vardy, Nigel M. Hooper, David M. Mann, Stuart Pickering-Brown, Kristelle Brown, James Lowe, Kevin Morgan, A. David Smith, Gordon Wilcock, Donald Warden, Clive Holmes, Henne Holstege, Eva Louwersheimer, Wiesje M. van der Flier, Philip Scheltens, John C. Van Swieten, Isabel Santana, Catarina Oliveira, Kevin Morgan, John F. Powell, John S. Kauwe, Carlos Cruchaga, Alison M. Goate, Andrew B. Singleton, Rita Guerreiro, John Hardy

**Affiliations:** a Reta Lila, Weston Research Laboratories, Department of Molecular Neuroscience, UCL Institute of Neurology, London, UK; b Laboratory of Neurogenetics, National Institute on Aging, National Institutes of Health, Bethesda, MD, USA; c Department of Experimental Neurology, Center for Stroke Research Berlin (CSB), Charite’ Universitätmedizin, Berlin, Germany; d German Center for Neurodegenerative Diseases (DZNE), Berlin site, Germany; e Department of Biology, Brigham Young University, Provo, UT, USA; f King's College London, Institute of Psychiatry, Psychology and Neuroscience, London, UK; g QIMR Berghofer Medical Research Institute, Brisbane, Queensland, Australia; h Institute of Biomedical and Clinical Science, University of Exeter Medical School, Exeter, Devon, UK; i Translation Cell Sciences-Human Genetics, School of Life Sciences, Queens Medical Centre, University of Nottingham, Nottingham, UK; j Neurogenetics Laboratory, Center for Neuroscience and Cell Biology, University of Coimbra, Coimbra, Portugal; k Department of Neurology, Alzheimer Center, VU University Medical Center, Neuroscience Campus Amsterdam, Amsterdam, the Netherlands; l Department of Neurology, Erasmus Medical Centre, Rotterdam, the Netherlands; m Neurology Department, Centro Hospitalar e Universitário de Coimbra, Coimbra, Portugal; n Faculty of Medicine, Coimbra University, Coimbra, Portugal; o CNC—Center for Neuroscience and Cell Biology, University of Coimbra, Coimbra, Portugal; p Laboratory of Biochemistry, Faculty of Medicine, University of Coimbra, Coimbra, Portugal; q Department of Neuroscience, Brigham Young University, Provo, UT, USA; r Division of Biology and Biomedical Sciences, Washington University, St. Louis, MO, USA; s Icahn School of Medicine at Mount Sinai, Icahn Medical Institute, New York, NY, USA

**Keywords:** Alzheimer's disease (AD), Genome-wide association studies (GWASs), *ABCA7*, Whole exome sequencing (WES), Whole genome sequencing (WGS), Protective variant

## Abstract

Genome-wide association studies (GWASs) have been effective approaches to dissect common genetic variability underlying complex diseases in a systematic and unbiased way. Recently, GWASs have led to the discovery of over 20 susceptibility loci for Alzheimer's disease (AD). Despite the evidence showing the contribution of these loci to AD pathogenesis, their genetic architecture has not been extensively investigated, leaving the possibility that low frequency and rare coding variants may also occur and contribute to the risk of disease. We have used exome and genome sequencing data to analyze the single independent and joint effect of rare and low-frequency protein coding variants in 9 AD GWAS loci with the strongest effect sizes after *APOE* (*BIN1*, *CLU*, *CR1*, *PICALM*, *MS4A6A*, *ABCA7*, *EPHA1*, *CD33*, and *CD2AP*) in a cohort of 332 sporadic AD cases and 676 elderly controls of British and North-American ancestry. We identified coding variability in *ABCA7* as contributing to AD risk. This locus harbors a low-frequency coding variant (p.G215S, rs72973581, minor allele frequency = 4.3%) conferring a modest but statistically significant protection against AD (*p*-value = 0.024, odds ratio = 0.57, 95% confidence interval = 0.41–0.80). Notably, our results are not driven by an enrichment of loss of function variants in *ABCA7*, recently reported as main pathogenic factor underlying AD risk at this locus. In summary, our study confirms the role of *ABCA7* in AD and provides new insights that should address functional studies.

## 1 Introduction

Alzheimer's disease (AD) is the most common cause of progressive dementia in the elderly. Aging and genetic factors play a critical role for the disease development. Rare coding and fully penetrant mutations in *APP*, *PSEN1*, and *PSEN2* explain part of the AD autosomal-dominant cases. On the other hand, *APOE* ε4 allele and rare coding variants in *TREM2* represent the main risk factors for late-onset and apparently sporadic AD (Chartier-Harlin et al., 1994, Guerreiro et al., 2013). In the last 5 years, genome-wide association studies (GWASs) identified over 20 main risk loci influencing AD susceptibility (Harold et al., 2009, Hollingworth et al., 2011, Lambert et al., 2009, Lambert et al., 2013, Naj et al., 2011, Seshadri et al., 2010). Among these, 9 have been replicated by at least 2 independent GWASs and present the strongest effect sizes after *APOE* (*BIN1*, *CLU*, *CR1*, *PICALM*, *MS4A6A*, *ABCA7*, *EPHA1*, *CD33*, and *CD2AP*).

GWASs have been a successful strategy to identify loci associated to a common trait, shedding light on disease pathways and for AD these include the following: (1) immune response (*CR1*, *MSA4A/MSA7A*, *CD2AP*, *CD33*, *EPHA1*, and *ABCA7*); (2) vesicles trafficking (*PICALM* and *BIN1*); (3) lipid metabolism (*CLU* and *ABCA7*); and (4) amyloid beta peripheral clearance (*PICALM*, *BIN1*, *CD33*, and *ABCA7*; http://www.alzgene.org/). Nevertheless, the functional variant(s) within these risk loci have not yet been fully defined.

GWAS arrays tag common, low penetrant, and generally noncoding variants that likely exert a subtle regulatory effect (0.8 < odds ratio [OR] < 1.5) on a trait, affecting gene expression, CpG islands methylation and splicing, in *cis* or *trans* (Ramasamy et al., 2014, Visscher et al., 2012). Whereas, low frequency (1% < minor allele frequency [MAF] < 5%) and rare variants (MAF < 1%) with a modest penetrance remain mostly undetected either because they are not in the array or because, even with the implementation of imputation, the detection of variants with MAF < 2% is not sufficiently accurate. As an illustrative example, *APOE* GWAS hit maps to an intronic region and it is likely driven by the *APOE* ε4 allele, which is a common coding haplotype (rs429358, p.C130R and rs7412, p.R176R, MAF = 15%) in exon 4, that is not tagged by the custom genotyping arrays mostly used.

Recently, resequencing studies have been powerful strategies to bridge the gap between susceptibility loci identified and actual disease-modifying variant(s) (Beaudoin et al., 2013, Lohmueller et al., 2013, Rivas et al., 2011, Service et al., 2014).

Therefore, we have used exome and genome sequencing data (1) to identify rare and low-frequency coding variants in *BIN1*, *CLU*, *CR1*, *PICALM*, *MS4A6A*, *ABCA7*, *EPHA1*, *CD33*, and *CD2AP* and (2) to investigate their single independent and combined effect on AD susceptibility. Both the single-variant and the gene-based association tests confirmed *ABCA7* as susceptibility locus associated with AD. Importantly, although *ABCA7* loss of function (LoF) mutations (indels, nonsense, and splice-site mutations) have been recently reported as main mechanism increasing AD risk at this locus (Steinberg et al., 2015), our results are not driven by such variants. Whereas, we report an enrichment for *ABCA7* common and low-frequency coding variants with a potential protective effect, that is mainly responsible for our gene-based signal. Among these, *ABCA7* p.G215S is the main low-frequency missense hit in the single-variant analysis in the discovery cohort. The potential protective role of this variant has been further confirmed in an independent European and North-American cohort. Our results show that *ABCA7* p.G215S exerts a mild but statistically significant influence, lowering the risk for AD. Thus, confirming *ABCA7* to be a good potential target to address functional studies.

## 2 Materials and methods

The discovery cohort was composed of 332 apparently sporadic AD cases and 676 elderly controls, neuropathologically and clinically confirmed, originating from the UK and North America. The mean age at disease onset was 71.66 years (range 41–94 years) for cases and the mean age of ascertainment was 78.15 years (range 60–102 years) for controls (Table 1). Most of the AD cases (77%) were late onset (>65 years at onset) (LOAD).

**Fig. 1:**
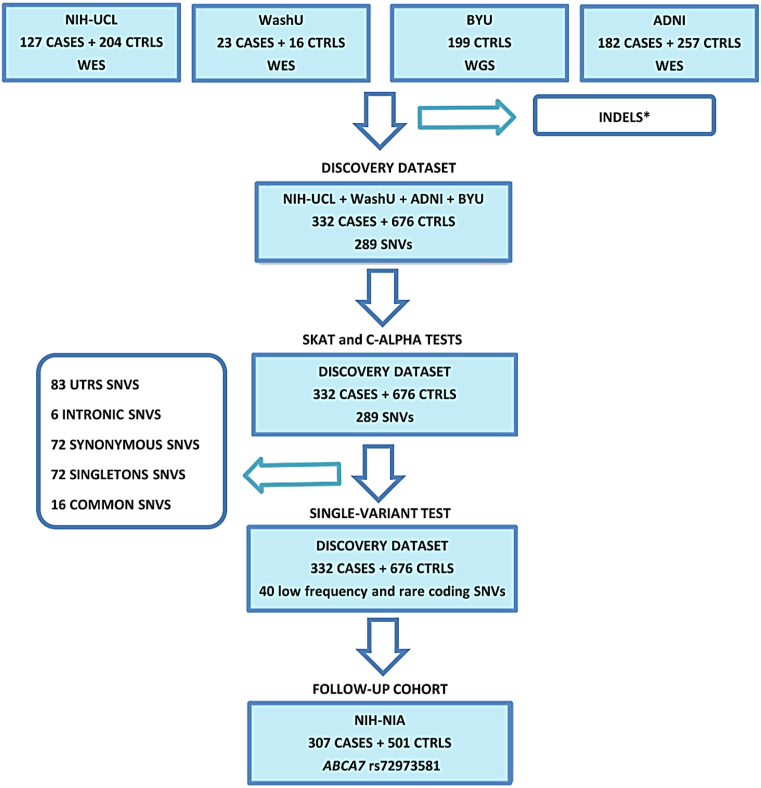
Pipeline of our study design. ^∗^INDELS have been excluded from the discovery cohort because not targeted in the ADNI data set. Abbreviations: ADNI, Alzheimer's Disease Neuroimaging Initiative; CTRLS, controls; INDELS, in-frame insertions and deletions; SNVs, single-nucleotide variants; UTRs, untranslated regions; WES, whole exome sequencing; WGS, whole genome sequencing.

Among the cases and controls, 42% and 51% were female, respectively; 58% and 47% of the cases and controls carried the *APOE* ε4 allele, respectively. The *APOE* ε4 allele was significantly associated to the disease status in the National Institutes of Health (NIH) and Alzheimer's Disease Neuroimaging Initiative (ADNI) series (*p*-value = 0.02 and 1.19 × 10^−9^, respectively). The threshold call rate for inclusion of the subject in analysis was 95%. On this cohort, we performed (1) gene-based analysis (SNP-set Sequence Kernel Association Test [SKAT] and c-alpha tests) and (2) single-variant association analysis, targeting 23.5 kilobase pairs (Kbs) of coding sequence. Finally, we followed up, in an independent Caucasian data set, *ABCA7* p. G215S, the only nominal significant low-frequency missense variant in the single-marker association test in our discovery set (Fig. 1).

The follow-up data set was composed of 307 late-onset apparently sporadic AD cases from North America and Europe and 501 elderly Caucasian controls from North America (Coriell repositories), Europe, Australia, and Canada (Table 1). Written informed consent was obtained for each clinically assessed individual, and the study was approved by the appropriate institutional review boards. All samples had fully informed consent for retrieval and were authorized for ethically approved scientific investigation (UCLH Research Ethics Committee number 10/H0716/3, BYU IRB, Cardiff REC for Wales 08/MRE09/38+5, REC Reference 04/Q2404/130, National Research Ethics Service).

### 2.1 Exome sequencing

DNA was extracted from blood or brain for cases and brain only for controls using standard protocols. Library preparation for next generation sequencing used DNA (between 1 μg and 3 μg) fragmented in a Covaris E210 (Covaris Inc). DNA was end-repaired by 5'phosphorylation, using the Klenow polymerase. A polyadenine tail was added to the 3'end of the phosphorylated fragment and ligated to Illumina adapters. After purification using an AMPure DNA Purification kit (Beckman Coulter, Inc), adapter-ligated products were amplified. The DNA library was then hybridized to an exome capture library (NimbleGen SeqCap EZ Exome v2.0, Roche Nimblegen Inc or TruSeq, Illumina Inc) and precipitated using streptavidin-coated magnetic beads (Dynal Magnetic Beads, Invitrogen). These exome libraries were polymerase chain reaction amplified and then DNA hybridized to paired-end flow cells using a cBot (Illumina, Inc) cluster generation system. Samples were sequenced on the Illumina HiSeq 2000 using 2 × 100 paired-end reads cycles.

### 2.2 Whole genome sequencing

Genome sequencing was performed in 199 controls, from the Cache County Study on Memory in Aging. All samples were sequenced with the use of Illumina HiSeq technology.

### 2.3 Sanger sequencing

*ABCA7* p.G215S (rs72973581) was screened in an additional follow-up cohort composed of 307 late-onset AD cases and 501 elderly controls. Primers were designed in Primer3 (http://bioinfo.ut.ee/primer3-0.4.0/) using the University California Santa Cruz ​(UCSC) (http://genome.ucsc.edu/) reference sequences NM_019112 (*ABCA7*).

Purified sequences were analyzed on an ABI 3730 DNA Analyzer (Applied Biosystems, CA, USA) and chromatograms were visualized in Sequencher software (version 4.2 Gene Codes Corporation, MI, USA).

### 2.4 Bioinformatics

Sequence alignment and variant calling were performed against the reference human genome (UCSC hg19). Alignment was performed with the use of CASAVA software, and variant calling was performed with the use of SAMtools (Li et al., 2009) and the Genome Analysis Toolkit (GATK) (McKenna et al., 2010). Paired-end sequence reads (2 × 100 bp paired-end read cycles) were aligned using the Burrows-Wheeler aligner (Li and Durbin, 2009). Format conversion and indexing were performed with Picard (www.picard.sourceforge.net/index.shtml). GATK was used to recalibrate base quality scores, perform local realignments around indels, and to call and filter the variants (McKenna et al., 2010). VCFtools was used to annotate gene information for the remaining novel variants. We used ANNOVAR software to annotate the variants (Wang et al., 2010). Variants were checked against established databases (1000 Genomes Project and dbSNP v.134). Calling algorithms, pipelines, and reference panels were the same as the pooled data sets. The protein coding effects of variants were predicted using SIFT, Polyphen2, and SeattleSeq Annotation (gvs.gs.washington.edu/SeattleSeqAnnotation). All variants within the coding regions of the 9 risk loci (*ABCA7* [NM_019112]; *CD2AP* [NM_012120]; *MS4A6A* [NM_152851]; *CR1* [NM_000573]; *BIN1* [NM_139343]; *PICALM* [NM_001206946]; *EPHA1* [NM_005232]; *CLU* [NM_001831]; and *CD33* [NM_001772]) have been collected and analyzed. Indels were excluded from the merged data set because they were not targeted in the ADNI subcohort (Fig. 1) (Further details are provided in the Supplementary Materials).

### 2.5 Statistical analysis

In the single-variant analysis, allele frequencies were calculated for each low frequency and rare coding variant in cases and controls, and Fisher's exact test on allelic association was performed. To study the joint effect of the variants detected, we performed a gene-based analysis with SKAT and c-alpha test, and we analyzed together for each gene the whole spectrum of allelic variability (common, low frequency, rare, coding, and noncoding).

C-alpha test and SKAT are closely related, being both nonburden tests, analyzing and collapsing the effect of genetic variants of different frequency (common and rare), effect (protective, damaging, and neutral), and effect size (modest, moderate, and strong). SKAT can be considered an expansion of the c-alpha test because it overcomes some of its limits. Indeed, SKAT (1) can be applied also to the study of continuous traits and (2) does not need any permutation.

Low frequency and rare variants were defined as having a 1% < MAF < 5% and MAF <1%, respectively, either in cases or controls.

All computations, c-alpha, and SKAT tests were performed in R (version ×64 3.0.2, http://www.r-project.org/) and PLINK/SEQ.

A *p*-value of 0.05 was set as a nominal significance threshold. Based on multiple testing correction, the thresholds for single-variant and gene-based association tests are defined by *p*-value = 1.25 × 10^−3^ (0.05/40 [total number of coding low frequency and rare variants detected in our study]) and 5.5 × 10^−3^ (0.05/9 genes), respectively. Furthermore, we excluded singletons from the single-variant analysis because a variant observed only once is not largely informative about the overall distribution (Neale et al., 2011). However, we pooled the singletons together and analyzed their collective effect in the gene-based analysis (SKAT and c-alpha test).

In addition, we report the complete list of coding variants detected in these GWAS loci in the supplementary table (Supplementary Table 1).

## 3 Results

The discovery set consisted of a total of 332 sporadic and mainly late-onset AD cases and 676 elderly controls of British and North-American ancestry (Table 1).

A total of 289 single-nucleotide variants were identified. Among these, 128 (44.3%) were nonsynonymous, 72 (24.9%) synonymous, 83 (28.7%) were untranslated region (UTR) and 6 (2%) intronic variants. Among the missense variants, 99 (77.34%) were rare (MAF < 1%) and 72 of these (72.72%) were singletons (a variant observed only once either in cases or controls). Fifteen nonsynonymous variants (11.7%) were low frequency (1% < MAF < 5%) and 16 (12.5%) were common (MAF ≥ 5%). In addition, we report 14 novel coding variants (not reported in ExAC, released 13 January 2015, or dbSNP 137). None of the detected low frequency and rare coding variants clusters within common haplotypes (MAF ≥ 5%) and, therefore, could have been missed by GWASs and chip based fine-mapping approaches (Supplementary Tables 1 and 2). We report the presence of 2 or 3 low frequency and/or rare variants in the studied genes in the same individual, both for cases and controls (Supplementary Table 3a and b).

Overall, the total variant frequency of the 9 GWAS loci in our study was in line with the one reported for the European-American cohort in the Exome Variant Server (http://evs.gs.washington.edu/EVS/). The only exception was represented by *CR1*, that showed a 2.7-fold higher relative frequency of total variants, compared to the Exome Variant Server database (Supplementary Table 4).

*PICALM* harbors the lowest burden of low frequency and rare coding variants (3.27 coding variants per kb of coding sequence). By contrast, *CD33*, presents the highest relative frequency of coding variants and the lowest relative frequency of damaging variants (9.14 and 0.91 coding and damaging variants per kb of coding sequence, respectively), suggesting that most coding variability in *CD33* is likely nonfunctional (Supplementary Table 5).

*BIN* and *ABCA7* display the highest relative proportion of damaging variants (3.92 [87.3% of its coding variability] and 3.72 [60% of its coding variability] damaging coding variants per kb of coding sequence, respectively), thus arguing for a potential functional impact of missense mutations at these loci (Supplementary Table 5). Moreover, *ABCA7* was the only gene harboring nonsense mutations.

Most of the low frequency and rare coding variability identified within these loci exerts generally a relatively modest effect (mean OR = 1.1) that is comparable to those observed for common and generally noncoding variants identified by GWASs (Supplementary Table 1).

### 3.1 Single coding variant association test

The main hits of the single variants association test map mainly to *ABCA7* (Table 2). Particularly, we report *ABCA7* p.G215S (rs72973581), that was the only low-frequency (MAF = 4.3%) missense variant showing a trend toward significance in the single-marker association test (*p*-value = 0.02 and corrected *p*-value = 0.8) in the discovery set and was statistically significant after Bonferroni correction (*p*-value = 6 × 10^−4^ and corrected *p*-value = 0.024) in the combined data sets (discovery set and follow-up data set).

Rs72973581 [A] results in a glycine to serine amino acid change at the position 215 of ATP-binding cassette subfamily A member 7 (ABCA7; G215S) and its frequency was 1.56-fold higher in controls compared to cases (MAF = 4.66% and 7.24% for cases and controls, respectively), arguing for a protective effect (OR = 0.6, 95% confidence interval [CI] = 0.38–0.95). This variant was present in homozygosity in one control. The study possessed relatively low power to detect a significant association between cases and controls for low frequency and rare variants. Therefore, we have followed up *ABCA7* p.G215S, carrying out Sanger sequencing in an independent data set composed of 307 Caucasian late-onset AD cases and 501 elderly Caucasian controls (*p*-value = 0.012; OR = 0.54, 95% CI 0.31–0.89). In this follow-up data set, we confirmed a higher frequency of the *ABCA7* p.G215S variant in controls compared to cases (carrier frequency = 13.5% vs. 7.8% [1.7-fold] and MAF = 7% vs. 4.3% [1.6-fold], respectively).

Finally, we also report a common coding polymorphism in *ABCA7* (p.R1349Q, rs3745842), that maps 1.3 kb from a reported GWAS hit, rs3752246, but clusters within a different common haplotype block (MAF > 5%) (Naj et al., 2011; Supplementary Table 2). Rs3745842 major allele (G) was more frequent in cases compared to controls, although the association was nominally significant after multiple testing correction (*p*-value = 1.4 × 10^−3^, corrected *p*-value = 0.081) (Supplementary Table 6).

### 3.2 LoF mutations in *ABCA7*

LoF mutations in *ABCA7* have been recently reported as main mechanism explaining the GWAS signal and the increased susceptibility to AD.

In our cohort, we detected 5 LoF mutations in *ABCA7*: 2 stopgain mutations (p.Y1579X and p.E1974X) and 3 splice-site or near splice-site mutations (c.7-2A>G, c.7-7T>C and c.231-12C>A). *ABCA7* p.E1974X and c.231-12C>A are novel variants and, together with p.Y1579X, are singletons, detected only in controls (Supplementary Table 7).

Importantly, the enrichment for *ABCA7* LoF mutations and novel variants in controls did not rely on the sequencing strategy (exome sequencing vs. genome sequencing). These variants have not been indeed mainly detected in the 199 BYU controls that underwent genome sequencing (Supplementary Table 8).

Moreover, 3 very rare indels have been identified in controls in the NIH-UCL cohort (p.1402delT, p.1638delCTT, and p.1749delCTACTG). *ABCA7* p.1749delCTACTG is a novel mutation, and *ABCA7* p.1402delT was also present in one case. These indels have been excluded from the pooled data set because they are not targeted in the ADNI subcohort (Supplementary Table 9; Fig. 1).

Finally, 3 nonsense mutations (p.W749X, p.W903X, and p.R1754X) and one splice-site mutation, (c.4416+2T>G) did not pass either the sample or variant quality control (QC) criteria and, therefore, have not been included in the study (Supplementary Table 10).

### 3.3 Gene-based association test

In addition to single-marker analysis, we carried out gene-wide analysis to combine the joint signal from multiple variants (coding variants and flanking UTRs) within a gene and to provide greater statistical power than that for single-marker tests. All the variants (nonsynonymous, synonymous, UTRs, and singletons) located within the studied genes and their exon-intron flanking regions were collapsed together and their combined effect was studied. *ABCA7* was the main hit both in the SKAT and c-alpha test, nominally and statistically significant, respectively, after multiple testing correction (corrected *p*-value = 0.6 and 5.3 × 10^−3^, respectively) (Table 3, Table 4). Importantly, given the exclusion of indels in the merged discovery data set, the presence of only 2 nonsense singleton mutations in *ABCA7* (p.Y1579X and p.E1974X, both detected in controls) and 4 putative splice-site mutations (rs3752229, rs2242437, c.231–12C>A, and rs182233998, the latter one nominally significant in controls), our findings are not influenced by a burden of LoF mutations in *ABCA7*. Considering the very rare frequency of these LoF variants, their detection with sufficient power would have required a very large sample size. By contrast, the top signals are represented mainly by common and low-frequency coding variants with a higher frequency in controls compared to cases and with a modest to intermediate protective effect (0.329 < OR < 0.755) (Supplementary Table 6).

## 4 Discussion

We report the results of single-variant and gene-based association tests performed in *BIN1*, *CLU*, *CR1*, *PICALM*, *MS4A6A*, *ABCA7*, *EPHA1*, *CD33*, and *CD2AP* in a cohort composed of 332 apparently sporadic and mainly late-onset AD cases and 676 elderly Caucasian controls from North America and the UK. In the single-variant association test, we have analyzed the effect of low frequency and rare coding variants (MAF < 5%), aiming to identify potential functional variant(s) underlying the GWAS hit(s). In the gene-based analysis (SKAT and c-alpha test), we collapsed the full spectrum of variants identified in these loci to study their collective effect.

We do not report any pathogenic mutation in *APP*, *PSEN1*, and *PSEN2* in our cohort. However, one of the controls was a heterozygous carrier of the protective variant *APP* p.A673T (MAF 7 × 10^−4^ in our cohort and MAF 5 × 10^−4^ among the European non-Finnish, ExAC database, released 13 January, 2015) (Jonsson et al., 2012).

*TREM2* p.R47H, the second most common risk factor for sporadic AD, has been detected in 6 cases (1.8%) and 4 controls (0.59%) and, likely given our small sample size, with a MAF = 0.2%, was not significantly associated to AD (*p*-value = 0.09).

*ABCA7* was the only significant hit in the c-alpha test and harbors a low-frequency coding variant (p.G215S, rs72973581), whose minor allele confers a modest (OR = 0.57, 95% CI = 0.41–0.80) but statistically significant protection (corrected *p*-value =0.024) against AD. Importantly, this single-nucleotide polymorphism (SNP) was not present in several GWAS or exome SNP arrays and does not cluster within common haplotypes identified by tagging SNPs, whereas it has been detected through *ABCA7* direct sequencing in the present study. Therefore, rs72973581 would have stayed likely undetected using common fine-mapping genotyping arrays. In addition, it does not cluster in the risk haplotypes identified by GWAS main hits (rs3764650, rs115550680, rs3752246, and rs4147929), suggesting an independent signal and a likely different pathogenic mechanism of the major allele (Hollingworth et al., 2011, Liu et al., 2014, Naj et al., 2011, Reitz et al., 2013). Importantly, the *ABCA7* p.G215S significant protective role against AD is supported by a targeted resequencing study of *ABCA7* in a Belgian cohort, where rs72973581 (A) frequency was 1.34-fold higher in controls compared to cases (*p*-value = 0.055) (Cuyvers et al., 2015). Notably, the main variant associated to LOAD in this Belgian cohort was a low-frequency intronic variant (rs78117248) that did not pass our QC filter. However, in line with our findings, Cuyvers et al. report an enrichment for common and low-frequency polymorphism with a modest protective role in *ABCA7*. Importantly, among the top 10 genetic variants identified in our study, 3 missense mutations (rs74176364, rs114782266, and rs117187003) have been described associated also to autism spectrum disorder, strongly pointing toward a functional role of these amino acid changes and suggesting a possible shared pathogenic mechanisms underpinning neurodegenerative and neurodevelopmental diseases (He et al., 2014).

Interestingly, several lines of evidence reported that a significant decrease in ABCA7 levels is associated to AD. At this regard, different and likely not mutually exclusive mechanisms have been described to influence the protein level: (1) common and generally noncoding variants in regulatory regions; (2) alternative splicing; (3) increased CpG island methylation (Humphries et al., 2015, Vasquez et al., 2013). Recently, also LoF mutations in *ABCA7* have been shown to significantly increase the susceptibility to AD in the Islandic population (Steinberg et al., 2015). This has been replicated in 2 different populations (Caucasian North American and Belgian) by 2 independent studies (Cuyvers et al., 2015, Vardarajan et al., 2015). Therefore, we report another potential mechanism, through which low-frequency protein coding variability in *ABCA7* may influence AD risk.

Notably, *ABCA7* p.G215S provides critical insights into the genetic architecture of diseases, reinforcing the view that GWAS loci, likewise Mendelian genes, harbor low frequency and rare protective coding variants that can counteract with a similar effect size the damaging alleles (OR ≈0.6 vs. ≈1.1 and ≈0.2 vs. ≈5, for GWAS loci and Mendelian genes, respectively; Asante et al., 2015, Jonsson et al., 2012, Nejentsev et al., 2009, Rivas et al., 2011).

*ABCA7* is mainly expressed in leukocytes and in myelolymphatic tissues (thymus, spleen, and bone marrow) and microglia in the brain (http://web.stanford.edu/group/barres_lab/brain_rnaseq.html; http://www.uniprot.org/; Kim et al., 2008). *ABCA7* encodes for ABCA7, a multi-pass protein, present on the cell, Golgi, and endosome membranes (http://www.uniprot.org/). In vitro and in vivo experiments have shown ABCA7 pivotal role in phagocytosis and a likely modest role in high-density lipoprotein biogenesis. In *Abca7*^−/−^ mice, macrophages and microglia display impaired phagocytosis and clearance of amyloid from the brain, which leads to cognitive impairment (Iwamoto et al., 2006, Tanaka et al., 2011).

Therefore, *ABCA7*, likewise *TREM2* and *CD33*, may play an important role in regulating microglial uptake and clearance of amyloid-beta debris.

*ABCA7* p.G215S clusters within the extracellular topological domain of ABCA7. Remarkably, at the homologous residue, the serine is the reference amino acid in ABCA7 in different mammals and in the homologous protein ABCA1 in humans. Thus, suggesting that this amino acid may confer some biologic advantage and may have been positively selected during the evolution (Supplementary Figs. 1 and 2).

Likewise other low-frequency and rare protective variants at the GWAS loci (Supplementary Table 11), *ABCA7* p.G215 is a relatively conserved residue among different species (Supplementary Fig. 1) and this amino acid change (glycine to serine) may only slightly modify the protein activity (−5.86, 56, Gerd and Grantham score, respectively). Moreover, it has been reported as a tolerated change and benign, arguing against any possible LoF or significant impairment of ABCA7, that has been indeed associated to increased risk for AD (Steinberg et al., 2015). The biological effect of this substitution may therefore lead to a mild *ABCA7* gain of function, possibly strengthening the interaction with a binding protein or regulating its expression. Although *ABCA7* p.G215 has not been predicted to be a coding target for miRNA (https://www.umm.uni-heidelberg.de), a possible posttranscriptional or posttranslational regulation should not be excluded. Importantly, the substitution of a glycine with a serine may imply an additional substrate for serine-kinases or proteases. Moreover, in a similar way, *ABCA1*, whose LoF variants have been associated to AD (Kim et al., 2012, Nordestgaard et al., 2015), has been reported to be particularly enriched for low frequency and rare coding variants with an average 1.5-fold higher frequency in controls compared to LOAD cases and a modest protective effect in a Greek cohort (OR = 0.96–0.38; Lupton et al., 2014; Supplementary Table 12).

Thus, understanding the effect of *ABCA7* p.G215S has the potential of unraveling new pathogenic mechanisms underpinning AD and may provide a promising therapeutic target that would not significantly alter ABCA7 overall physiological function, which is critical for AD development.

Finally, we support the resequencing study of the GWAS loci by Vardarajan et al. (2015), confirming a burden of damaging variants in *ABCA7* and *BIN1* (Supplementary Table 5) and to a lesser extent in *CD2AP*, *EPHA1*, and *MS4A6A* (main hits in the gene-based analysis; Table 3, Table 4), highlighting their potential role as susceptibility loci for LOAD.

However, we could not replicate the main hits detected by Vardarajan et al. in the single-variant analysis, either because such variants have been targeted but not detected in our cohort (*ABCA7* p.E1679X, *EPHA1* p.P460L, and *BIN1* p.K358R) or because the variants have been targeted but eliminated by the QC filter (*CD2AP* p.K633R). Thus, suggesting a possible lack of replication compared to the previous studies attributable to the different population but also different sequencing strategies, capture, and coverage. Nevertheless, *EPHA1* and *CD2AP* harbor 2 of the main hits detected in the single-variant analysis in our cohort (rs11768549 and rs143297472, respectively) (Table 2), with rs11768549 already associated with the rapid progression of the disease in a cohort of Caucasian North American LOAD cases (Wang et al., 2015).

In summary, we support previous studies, suggesting that (1) *ABCA7* significantly influences AD risk; (2) *ABCA7* p.G215S is likely to reduce the susceptibility to AD; (3) GWAS hits are pleomorphic loci harboring a complex spectrum of variants synergistically contributing to the disease phenotype with different mechanisms, effects (damaging, protective, and neutral), and effect sizes (0 < OR < 4); and (4) gene-based approaches are effective methods to mine genetic data and to accurately filter potential candidate genes.

## Disclosure statement

The authors declare no competing financial or personal interests that can influence the presented work.

## Figures and Tables

**Table 1:** Description of the different cohorts used in this study

Cohorts	N	Type	Sequencing strategy	Origin	Age (y), Mean ± SD (range)	Male (%)	*APOE*, E4+ (%)
Discovery set							
NIH-UCL							
Cases	127	Neuropath	Exome sequencing	Caucasian (British)	65.5 (41–94)	46.4	58
Controls	204	Neuropath	Exome sequencing	Caucasian (British, North American)	79.8 (61–102)	58.3	45
WashU							
Cases	23	Clinical	Exome sequencing	Caucasian (North American)	57 (46–75)	52.17	NA
Controls	16	Clinical	Exome sequencing	Caucasian (North American)	79.5 (75–92)	43.7	NA
ADNI							
Cases	182	Clinical	Exome sequencing	Caucasian (North American)	74.65 (55–90)	67	56.6
Controls	257	Clinical	Exome sequencing	Caucasian (North American)	74.68 (60–90)	50.1	27.6
BYU							
Controls	199	Clinical	Genome sequencing	Caucasian (North American)	80.8 (75–94.5)	37.7	100
Follow-up genotyping set *ABCA7* rs72973581							
NIH-NIA							
Cases	307	Clinical	Sanger sequencing	Caucasian (North American, British, Dutch, Italian, Portuguese)	Average >65 y		
Controls	501	Clinical	Sanger sequencing	Caucasian, (North American, British, Greek, German, Polish, Australian, Canadian)	>60 y		

Key: *ABCA7*, ATP-binding cassette subfamily A member 7; N, number; NA, not applicable; UCL, University College London; WashU, Washington University; ADNI, Alzheimer's Disease Neuroimaging Initiative; BYU, Brigham Young University; NIA, National Institute on Aging; NIH, National Institutes of Health; SD, standard deviation.

**Table 2:** Most significant variants detected in our discovery set

Gene	Position	MA	cDNA change	Aa change	Rs	MAF cases-controls (%)	MAF ExAC (%)	SIFT	Polyphen	Mutation assessor	aa/Aa/AA cases	aa/Aa/AA controls	*p*-value	Corr. *p*-value	OR (95% CI)
*ABCA7*	19:1043103	A	c.G643A	p.G215S	rs72973581	4.66–7.24^a^	4.31^b^	Tolerated	Benign	Low	0/31/301	1/96/579	0.02	0.8	0.61 (0.38–0.95)
											0/55/584^c^	1/164/1012^c^	0.0006^c^	0.024	0.57^c^ (0.41–0.80)
*ABCA7*	19:1050996	A	c.G2629A	p.A877T^d^	rs74176364	0.3–1.18	1.69	Deleterious	Benign	Low	0/2/330	0/16/660	0.07	2.8	0.25 (0.02–1.07)
*EPHA1*	7:143095153	A	c.G1475A	p.R492Q^e^	rs11768549	2.56–1.47	1.21	Tolerated	Benign		0/17/315	1/18/657	0.07	2.8	1.86 (0.89–3.84)
*ABCA7*	19:1059056	A	c.G5435A	p.R1812H^d^	rs114782266	1.5–0.81	1.05	Tolerated	Benign	Neutral	0/10/322	0/11/665	0.16	6.4	1.87 (0.70–4.92)
*ABCA7*	19:1057343	A	c.G4795A	p.V1599M^d^	rs117187003	0.6–0.22	0.3	Deleterious	Possibly damaging	Medium	0/4/328	0/3/673	0.22	8.8	2.73 (0.45–18.7)
*CD2AP*	6:47573971	A	c.G1488A	p.M496I	rs143297472	0.3–0.07	NA	Tolerated	Benign		0/2/330	0/1/675	0.25	10	4.08 (0.21–241.3)
*ABCA7*	19:1047537	C	c.A2153C	p.N718T	rs3752239	1.65–2.44	7.02	Deleterious	Benign	Low	0/11/321	0/33/641	0.32	12.8	0.66 (0.29–1.37)

Position is in hg19/GRCh37.

Key: cDNA, complementary DNA; CI, confidence interval; Corr, corrected *p*-value, *p*-value after Bonferroni correction (*p*-value∗ 40 [number of variants considered in the single-variant association test]); ExAC, Exome Aggregation Consortium; ​MA, minor allele; MAF, minor allele frequency; ExAC, Exome Aggregation Consortium; OR, odds ratio.

a MAF cases-controls reported a Belgian cohort = 4.66%–6.27% (Cuyvers et al., 2015).

b MAF in ExAC (European non-Finnish) = 6.14% and MAF in EVS (European American) = 6.24%.

c Combined results discovery and follow-up data set.

d Variants reported associated also with autism spectrum disorders (ASD) (He et al., 2014).

e Variant reported associated to a more rapid disease progression.

**Table 3:** Results from the c-alpha test performed

Transcript ID	Position	Gene	N.variants	Test	*p*-value	Corrected *p*-value
NM_019112	chr19:1040131…1065563	*ABCA7*	72	c-alpha	0.0006	0.0053
NM_012120	chr6:47445789…47594915	*CD2AP*	20	c-alpha	0.0353	0.31
NM_152851	chr11:59939123…59950523	*MS4A6A*	11	c-alpha	0.0548	0.49
NM_000573	chr1:207669709…207814864	*CR1*	72	c-alpha	0.0677	0.6
NM_139343	chr2:127805799…127864546	*BIN1*	27	c-alpha	0.0730	0.65
NM_001206946	chr11:85668697…85779900	*PICALM*	19	c-alpha	0.0742	0.66
NM_005232	chr7:143088365…143105830	*EPHA1*	30	c-alpha	0.1065	0.95
NM_001831	chr8:27454493…27472251	*CLU*	29	c-alpha	0.4444	3.99
NM_001772	chr19:51728380…51743144	*CD33*	13	c-alpha	0.7142	6.42

Position is in hg19/GRCh37. Corrected *p*-value, *p*-value after Bonferroni correction (*p*-value^*^ 9 [number of genes considered in the single gene-based analysis]).

**Table 4:** Results from the SKAT test performed

Transcript ID	Position	Gene	N.variants	Test	*p*-value	Corrected *p*-value
NM_019112	chr19:1040131…1065563	*ABCA7*	72	SKAT	0.0737	0.66
NM_005232	chr7:143088365…143105830	*EPHA1*	30	SKAT	0.2981	2.68
NM_139343	chr2:127805799…127864546	*BIN1*	27	SKAT	0.4472	4.02
NM_012120	chr6:47445789…47594915	*CD2AP*	20	SKAT	0.4489	4.04
NM_000573	chr1:207669709…207814864	*CR1*	72	SKAT	0.5105	4.59
NM_001831	chr8:27454493…27472251	*CLU*	29	SKAT	0.5902	5.31
NM_152851	chr11:59939123…59950523	*MS4A6A*	11	SKAT	0.9377	8.43
NM_001772	chr19:51728380…51743144	*CD33*	13	SKAT	0.9389	8.45
NM_001206946	chr11:85668697…85779900	*PICALM*	19	SKAT	0.9437	8.49

Position is in hg19/GRCh37. Corrected *p*-value, *p*-value after Bonferroni correction (*p*-value^*^ 9 [number of genes considered in the single gene-based analysis]).
