# Supplementary material for: ABCA7 p.G215S as potential protective factor for Alzheimer's disease
Source: Neurobiol Aging. 2016 Oct;46:235.e1–9. doi: 10.1016/j.neurobiolaging.2016.04.004 (PMC5024078; doi:10.1016/j.neurobiolaging.2016.04.004)
Supplement: Supplementary Materials [file mmc1.docx]

| **GENE** | **POSITION** | **cDNA change** | **Aa change** | **rs ID** | **MAF** | **ExAC** | **MA** | **SIFT** | **POLYPHEN** | **aa/Aa/AA cases** | **aa/Aa/AA ctrls** | **P-VALUE** | **OR** |
| --- | --- | --- | --- | --- | --- | --- | --- | --- | --- | --- | --- | --- | --- |
|  |  |  |  |  | **cases-ctrls** |  |  |  |  |  |  |  |  |
| ***ABCA7*** | 19:1043103 | c.G643A | p.G215S | [rs72973581](http://browser.1000genomes.org/Homo_sapiens/Variation/Summary?db=core;g=ENSG00000064687;r=19:1040102-1065571;t=ENST00000263094;v=rs72973581;vf=17062840;source=dbSNP) | 0.04669-0.07249 | 0.04316 | A | Tolerated | Benign | 0/31/301 | 1/96/579 | 0.026 | 0.615 |
| ***ABCA7*** | 19:1050996 | c.G2629A | p.A877T | [rs74176364](http://browser.1000genomes.org/Homo_sapiens/Variation/Summary?db=core;g=ENSG00000064687;r=19:1040102-1065571;t=ENST00000263094;v=rs74176364;vf=17955633;source=dbSNP) | 0.003012-0.01183 | 0.01692 | A | Deleterious | Benign | 0/2/330 | 0/16/660 | 0.072 | 0.25 |
| ***ABCA7*** | 19:1059056 | c.G5435A | p.R1812H | [rs114782266](http://browser.1000genomes.org/Homo_sapiens/Variation/Summary?db=core;g=ENSG00000064687;r=19:1040102-1065571;t=ENST00000263094;v=rs114782266;vf=27445388;source=dbSNP) | 0.01506-0.008136 | 0.01057 | A | Tolerated | Benign | 0/10/322 | 0/11/665 | 0.162 | 1.87 |
| ***ABCA7*** | 19:1057343 | c.G4795A | p.V1599M | [rs117187003](http://browser.1000genomes.org/Homo_sapiens/Variation/Summary?db=core;g=ENSG00000064687;r=19:1040102-1065571;t=ENST00000263094;v=rs117187003;vf=29155372;source=dbSNP) | 0.006024-0.002219 | 0.003085 | A | Deleterious | Probably damaging | 0/4/328 | 0/3/673 | 0.226 | 2.73 |
| ***ABCA7*** | 19:1047537 | c.A2153C | p.N718T | [rs3752239](http://browser.1000genomes.org/Homo_sapiens/Variation/Summary?db=core;g=ENSG00000064687;r=19:1040102-1065571;t=ENST00000263094;v=rs3752239;vf=2823665;source=dbSNP) | 0.01657-0.02448 | 0.07028 | C | Deleterious | Benign | 0/11/321 | 0/33/641 | 0.325 | 0.665 |
| ***ABCA7*** | 19:1043794 | c.G1001A | p.R334Q | [rs147846250](http://browser.1000genomes.org/Homo_sapiens/Variation/Summary?db=core;g=ENSG00000064687;r=19:1040102-1065571;t=ENST00000263094;v=rs147846250;vf=38070265;source=dbSNP) | 0.001506-0 | 0.0001995 | A | Tolerated | Benign | 0/1/331 | 0/0/676 | 0.329 | Inf |
| ***ABCA7*** | 19:1044619 | c.C1091G | p.P364R | [rs146982710](http://browser.1000genomes.org/Homo_sapiens/Variation/Summary?db=core;g=ENSG00000064687;r=19:1040102-1065571;t=ENST00000263094;v=rs146982710;vf=37265489;source=dbSNP) | 0.001506-0 | 0.0003843 | G | Tolerated | Possibly damaging | 0/1/331 | 0/0/676 | 0.329 | Inf |
| ***ABCA7*** | 19:1046239 | c.C1456G | p.P486A | [rs141428162](http://browser.1000genomes.org/Homo_sapiens/Variation/Summary?db=core;g=ENSG00000064687;r=19:1040102-1065571;t=ENST00000263094;v=rs141428162;vf=32581564;source=dbSNP) | 0.001506-0 | 0.0003212 | G | Deleterious | Possibly damaging | 0/1/331 | 0/0/676 | 0.329 | Inf |
| ***ABCA7*** | 19:1047372 | c.G2062A | p.A688T | [rs376686030](http://browser.1000genomes.org/Homo_sapiens/Variation/Summary?db=core;g=ENSG00000064687;r=19:1040102-1065571;t=ENST00000263094;v=rs376686030;vf=64807385;source=dbSNP) | 0.001506-0 |  | A | Tolerated | Possibly damaging | 0/1/331 | 0/0/676 | 0.329 | Inf |
| ***ABCA7*** | 19:1053521 | c.C3414G | p.S1138R | rs1053521 | 0.001506-0 | 0.00002865 | G | Deleterious | Possibly damaging | 0/1/331 | 0/0/676 | 0.329 | Inf |
| ***ABCA7*** | 19:1056372 | c.T4460G | p.V1487G | rs200825702 | 0.001506-0 | 0.0001245 | G | Deleterious | Probably damaging | 0/1/331 | 0/0/676 | 0.329 | inf |
| ***ABCA7*** | 19:1056941 | c.G4622A | p.C1541Y | [rs145632609](http://browser.1000genomes.org/Homo_sapiens/Variation/Summary?db=core;g=ENSG00000064687;r=19:1040102-1065571;t=ENST00000263094;v=rs145632609;vf=36294516;source=dbSNP) | 0.001506-0 | 0.00004131 | A | Deleterious | Probably damaging | 0/1/331 | 0/0/676 | 0.329 | Inf |
| ***ABCA7*** | 19:1056958 | c.T4639C | p.S1547P | rs778244634 | 0.001506-0 | 0.00000826 | C | Deleterious | Probably damaging | 0/1/331 | 0/0/676 | 0.329 | Inf |
| ***ABCA7*** | 19:1057366 | c.G4818T | p.Q1606H | NOVEL | 0.001506-0 |  | T | Deleterious | Probably damaging | 0/1/331 | 0/0/676 | 0.329 | Inf |
| ***ABCA7*** | 19:1058655 | c.G5188A | p.V1730I | NOVEL | 0.001506-0 |  | A | Tolerated | Benign | 0/1/331 | 0/0/676 | 0.329 | inf |
| ***ABCA7*** | 19:1044712 | c.A1184G | p.H395R | [rs3764647](http://browser.1000genomes.org/Homo_sapiens/Variation/Summary?db=core;g=ENSG00000064687;r=19:1040102-1065571;t=ENST00000263094;v=rs3764647;vf=2834743;source=dbSNP) | 0.0256-0.03333 | 0.06298 | G | Tolerated | Benign | 0/17/315 | 0/45/630 | 0.403 | 0.755 |
| ***ABCA7*** | 19:1043748 | c.A955G | p.T319A | rs3752232 | 0.0256-0.03264 | 0.06039 | G | Tolerated | Benign | 0/17/315 | 0/44/630 | 0.403 | 0.77 |
| ***ABCA7*** | 19:1058176 | c.A5057G | p.Q1686R | [rs4147918](http://browser.1000genomes.org/Homo_sapiens/Variation/Summary?db=core;g=ENSG00000064687;r=19:1040102-1065571;t=ENST00000263094;v=rs4147918;vf=3193480;source=dbSNP) | 0.0256-0.0318 | 0.04785 | G | Tolerated | Benign | 0/17/315 | 0/43/633 | 0.481 | 0.794 |
| ***ABCA7*** | 19:1056109 | c.C4283T | p.S1428L | [rs145232000](http://browser.1000genomes.org/Homo_sapiens/Variation/Summary?db=core;g=ENSG00000064687;r=19:1040102-1065571;t=ENST00000263094;v=rs145232000;vf=35921262;source=dbSNP) | 0.001506-0.0007396 | 0.0001994 | T | Tolerated | Benign | 0/1/331 | 0/1/675 | 0.550 | 2.037 |
| ***ABCA7*** | 19:1053382 | c.T3275G | p.V1092G | [rs201213180](http://browser.1000genomes.org/Homo_sapiens/Variation/Summary?db=core;g=ENSG00000064687;r=19:1040102-1065571;t=ENST00000263094;v=rs201213180;vf=55225452;source=dbSNP) | 0.00303-0.0007452 | 0.0008056 | G | Deleterious | Possibly damaging | 1/0/329 | 0/1/670 | 0.550 | 2.034 |
| ***ABCA7*** | 19:1041922 | c.C253A | p.L85M | rs146597357 | 0-0.0007396 | 0.0002568 | A | Deleterious | Benign | 0/0/332 | 0/1/675 | 1 | 0 |
| ***ABCA7*** | 19:1041950 | c.T281C | p.L94P | NOVEL | 0-0.0007396 |  | C | Deleterious | Probably damaging | 0/0/332 | 0/1/675 | 1 | 0 |
| ***ABCA7*** | 19:1041971 | c.T302G | p.L101R | [rs201665195](http://browser.1000genomes.org/Homo_sapiens/Variation/Summary?db=core;g=ENSG00000064687;r=19:1040102-1065571;t=ENST00000263094;v=rs201665195;vf=55687437;source=dbSNP) | 0.001506-0.001479 | 0.0006849 | G | Deleterious | Possibly damaging | 0/1/331 | 0/2/674 | 1 | 1.018 |
| ***ABCA7*** | 19:1044708 | c.G1180C | p.G394R | rs781692277 | 0-0.0007396 | 0.000009175 | C | Deleterious | Possibly damaging | 0/0/332 | 0/1/675 | 1 | 0 |
| ***ABCA7*** | 19:1046944 | c.C1766G | p.A589G | [rs144979723](http://browser.1000genomes.org/Homo_sapiens/Variation/Summary?db=core;g=ENSG00000064687;r=19:1040102-1065571;t=ENST00000263094;v=rs144979723;vf=35573832;source=dbSNP) | 0-0.0007396 | 0.0007465 | G | Tolerated | Possibly damaging | 0/0/332 | 0/1/675 | 1 | 0 |
| ***ABCA7*** | 19:1047169 | c.T1859C | p.L620P | [rs144852598](http://browser.1000genomes.org/Homo_sapiens/Variation/Summary?db=core;g=ENSG00000064687;r=19:1040102-1065571;t=ENST00000263094;v=rs144852598;vf=35477502;source=dbSNP) | 0-0.001479 | 0.0003622 | C | Deleterious | Probably damaging | 0/0/331 | 0/2/674 | 1 | 0 |
| ***ABCA7*** | 19:1047336 | c.G2026A | p.A676T | [rs59851484](http://browser.1000genomes.org/Homo_sapiens/Variation/Summary?db=core;g=ENSG00000064687;r=19:1040102-1065571;t=ENST00000263094;v=rs59851484;vf=13839531;source=dbSNP) | 0-0.0007396 | 0.01294 | A | Deleterious | Probably damaging | 0/0/332 | 0/1/675 | 1 | 0 |
| ***ABCA7*** | 19:1049312 | c.C2428G | p.R810G | rs747154333 | 0-0.0007396 | 0.000008437 | G | Deleterious | Possibly damaging | 0/0/332 | 0/1/675 | 1 | 0 |
| ***ABCA7*** | 19:1049318 | c.C2434A | p.L812M | NOVEL | 0-0.0007396 |  | A | Deleterious | Probably damaging | 0/0/332 | 0/1/675 | 1 | 0 |
| ***ABCA7*** | 19:1056126 | c.C4300T | p.R1434C | [rs137888610](http://browser.1000genomes.org/Homo_sapiens/Variation/Summary?db=core;g=ENSG00000064687;r=19:1040102-1065571;t=ENST00000263094;v=rs137888610;vf=29665736;source=dbSNP) | 0-0.0007396 | 0.0001541 | T | Tolerated | Possibly damaging | 0/0/332 | 0/1/675 | 1 | 0 |
| ***ABCA7*** | 19:1057056 | c.C4737G | p.Y1579X | rs148266574 | 0-0.0007396 | 0.0000252 | G | NA | NA | 0/0/332 | 0/1/675 | 1 | 0 |
| ***ABCA7*** | 19:1058635 | c.C5168T | p.S1723L | rs73505232 | 0-0.0007396 | 0.01161 | T | Deleterious | Probably damaging | 0/0/332 | 0/1/675 | 1 | 0 |
| ***ABCA7*** | 19:1059029 | c.G5408A | p.R1803H | [rs143615723](http://browser.1000genomes.org/Homo_sapiens/Variation/Summary?db=core;g=ENSG00000064687;r=19:1040102-1065571;t=ENST00000263094;v=rs143615723;vf=34465542;source=dbSNP) | 0-0.0007396 | 0.000168 | A | Tolerated | Benign | 0/0/332 | 0/1/675 | 1 | 0 |
| ***ABCA7*** | 19:1063574 | c.T5744C | p.L1915P | NOVEL | 0-0.0007407 |  | C | Deleterious | Probably damaging | 0/0/332 | 0/1/674 | 1 | 0 |
| ***ABCA7*** | 19:1063651 | c.G5821A | p.V1941I | rs151130083 | 0-0.0007396 | 0.0004578 | A | Tolerated | Benign | 0/0/332 | 0/1/675 | 1 | 0 |
| **ABCA7** | 19:1056492 | c.G4580C | p.G1527A | [rs3752246](http://browser.1000genomes.org/Homo_sapiens/Variation/Summary?db=core;g=ENSG00000064687;r=19:1040102-1065571;t=ENST00000263094;v=rs3752246;vf=2823678;source=dbSNP) | 0.1767-0.1629 | 0.159 | C | Tolerated | Benign | 13/91/227 | 13/193/466 | 0.827 | 1.036 |
| ***ABCA7*** | 19:1063831 | c.G5920T | p.E1974X | NOVEL | 0-0.0007396 |  | T | NA | NA | 0/0/332 | 0/1/675 | 1 | 0 |
| **ABCA7** | 19:1042809 | c.A563G | p.E188G | [rs3764645](http://browser.1000genomes.org/Homo_sapiens/Variation/Summary?db=core;g=ENSG00000064687;r=19:1040102-1065571;t=ENST00000263094;v=rs3764645;vf=2834741;source=dbSNP) | 0.4517-0.4697 | 0.4838 | G | Tolerated | Benign | 67/165/99 | 154/327/195 | 0.768 | 0.950 |
| **ABCA7** | 19:1065018 | c.G6133T | p.A2045S | [rs4147934](http://browser.1000genomes.org/Homo_sapiens/Variation/Summary?db=core;g=ENSG00000064687;r=19:1040102-1065571;t=ENST00000263094;v=rs4147934;vf=3193496;source=dbSNP) | 0.4613-0.4163 | 0.268 | T | Tolerated | Benign | 114/70/139 | 179/179/287 | 0.680 | 1.061 |
| **ABCA7** | 19:1055191 | c.G4046A | p.R1349Q | [rs3745842](http://browser.1000genomes.org/Homo_sapiens/Variation/Summary?db=core;g=ENSG00000064687;r=19:1040102-1065571;t=ENST00000263094;v=rs3745842;vf=2815067;source=dbSNP) | 0.2987-0.385 | 0.4433 | A | Tolerated | Benign | 38/111/164 | 119/261/268 | 0.001 | 0.641 |
| ***BIN1*** | 2:127811524 | c.C1196T | p.P399L | rs558530329 | 0.001506-0 |  | T | Deleterious | Benign | 0/1/331 | 0/0/676 | 0.329 | Inf |
| ***BIN1*** | 2:127806110 | c.G1774T | p.V592F | [rs199908147](http://browser.1000genomes.org/Homo_sapiens/Variation/Summary?db=core;g=ENSG00000136717;r=2:127805603-127864931;t=ENST00000316724;v=rs199908147;vf=54036431;source=dbSNP) | 0-0.001479 | 0.0000165 | T | Deleterious | Probably damaging | 0/0/332 | 0/2/674 | 1 | 0 |
| ***BIN1*** | 2:127808064 | c.C1607A | p.T536K | rs773732601 | 0-0.0007396 | 0.0000165 | T | Deleterious | Possibly damaging | 0/0/332 | 0/1/675 | 1 | 0 |
| ***BIN1*** | 2:127808076 | c.C1595T | p.T532M | rs112318500 | 0-0.0007396 | 0.009155 | A | Deleterious | Possibly damaging | 0/0/332 | 0/1/675 | 1 | 0 |
| ***BIN1*** | 2:127808458 | c.T1492C | p.F498L | [rs368238742](http://browser.1000genomes.org/Homo_sapiens/Variation/Summary?db=core;g=ENSG00000136717;r=2:127805603-127864931;t=ENST00000316724;v=rs368238742;vf=56983602;source=dbSNP) | 0-0.0007396 | 0.00003322 | C | Tolerated | Benign | 0/0/332 | 0/1/675 | 1 | 0 |
| ***BIN1*** | 2:127825804 | c.C547T | p.P183S | NOVEL | 0-0.0007396 |  | T | Deleterious | Probably damaging | 0/0/332 | 0/1/675 | 1 | 0 |
| ***BIN1*** | 2:127808046 | c.A1625G | p.K542R | rs138047593 | 0.00753-0.01109 | 0.01122 | G | Deleterious | Probably damaging | 0/5/327 | 1/13/662 | 0.629 | 0.723 |
| ***BIN1*** | 2:127834212 | c.A155G | p.N52S | rs369549551 | 0.001506-0 | 0.000008237 | G | Tolerated | Probably damaging | 0/1/331 | 0/0/676 | 0.329 | Inf |
| ***CD2AP*** | 6:47573971 | c.G1488A | p.M496I | [rs143297472](http://browser.1000genomes.org/Homo_sapiens/Variation/Summary?db=core;g=ENSG00000198087;r=6:47445525-47594999;t=ENST00000359314;v=rs143297472;vf=34188888;source=dbSNP) | 0.003012-0.0007396 | | A | Tolerated | Benign | 0/2/330 | 0/1/675 | 0.253 | 4.084 |
| ***CD2AP*** | 6:47567069 | c.A1307G | p.K436R | NOVEL | 0.001506-0 |  | G | Deleterious | Probably damaging | 0/1/331 | 0/0/676 | 0.329 | Inf |
| ***CD2AP*** | 6:47563608 | c.A1120G | p.T374A | [rs138727736](http://browser.1000genomes.org/Homo_sapiens/Variation/Summary?db=core;g=ENSG00000198087;r=6:47445525-47594999;t=ENST00000359314;v=rs138727736;vf=30317910;source=dbSNP) | 0.00753-0.006657 | | G | Tolerated | Benign | 0/5/327 | 0/9/667 | 0.782 | 1.13 |
| ***CD2AP*** | 6:47512351 | c.A329C | p.K110T | NOVEL | 0-0.0007396 |  | C | Deleterious | Benign | 0/0/332 | 0/1/675 | 1 | 0 |
| ***CD2AP*** | 6:47541940 | c.C682T | p.R228W | [rs150851309](http://browser.1000genomes.org/Homo_sapiens/Variation/Summary?db=core;g=ENSG00000198087;r=6:47445525-47594999;t=ENST00000359314;v=rs150851309;vf=40535429;source=dbSNP) | 0-0.0007396 | 0 | T | Deleterious | Probably damaging | 0/0/332 | 0/1/675 | 1 | 0 |
| ***CD2AP*** | 6:47544775 | c.A839G | p.Y280C | rs765702919 | 0-0.0007396 |  | G | Deleterious | Benign | 0/0/332 | 0/1/675 | 1 | 0 |
| ***CD2AP*** | 6:47547209 | c.T992A | p.L331H | [rs140188898](http://browser.1000genomes.org/Homo_sapiens/Variation/Summary?db=core;g=ENSG00000198087;r=6:47445525-47594999;t=ENST00000359314;v=rs140188898;vf=31524572;source=dbSNP) | 0-0.0007396 | 0 | A | Tolerated | Benign | 0/0/332 | 0/1/675 | 1 | 0 |
| ***CD2AP*** | 6:47548605 | c.A1014G | p.P338P | NOVEL | 0-0.0007396 |  | G | NA | NA | 0/0/332 | 0/1/675 | 1 | 0 |
| ***CD33*** | 19:51728635 | c.A199G | p.I67V | rs746818326 | 0.001506-0 | 0.00004119 | G | Tolerated | Benign | 0/1/331 | 0/0/676 | 0.329 | Inf |
| ***CD33*** | 19:51728629 | c.G193C | p.A65P | [rs115684563](http://browser.1000genomes.org/Homo_sapiens/Variation/Summary?db=core;g=ENSG00000105383;r=19:51728320-51747115;t=ENST00000262262;v=rs115684563;vf=28093229;source=dbSNP) | 0-0.001479 | 0.003262 | C | Tolerated | Benign | 0/0/332 | 0/2/674 | 1 | 0 |
| ***CD33*** | 19:51728729 | c.G293A | p.R98K | rs148118239 | 0-0.0007396 | 0.001 | A | Tolerated | Benign | 0/0/332 | 0/1/675 | 1 | 0 |
| ***CD33*** | 19:51729594 | c.T727C | p.F243L | rs11882250 | 0-0.0007396 | 0.01017 | C | Tolerated | Benign | 0/0/332 | 0/1/675 | 1 | 0 |
| ***CD33*** | 19:51738465 | c.G799A | p.V267I | [rs58981829](http://browser.1000genomes.org/Homo_sapiens/Variation/Summary?db=core;g=ENSG00000105383;r=19:51728320-51747115;t=ENST00000262262;v=rs58981829;vf=13660415;source=dbSNP) | 0-0.0007396 | 0.01103 | A | Tolerated | Benign | 0/0/332 | 0/1/675 | 1 | 0 |
| ***CD33*** | 19:51738920 | c.T913C | p.S305P | [rs61736475](http://browser.1000genomes.org/Homo_sapiens/Variation/Summary?db=core;g=ENSG00000105383;r=19:51728320-51747115;t=ENST00000262262;v=rs61736475;vf=14332601;source=dbSNP) | 0.01355-0.01479 | 0.04688 | C | Tolerated | Benign | 0/9/323 | 0/20/656 | 1 | 0.914 |
| ***CD33*** | 19:51728815 | c.T379C | p.Y127H | [rs146181856](http://browser.1000genomes.org/Homo_sapiens/Variation/Summary?db=core;g=ENSG00000105383;r=19:51728320-51747115;t=ENST00000262262;v=rs146181856;vf=36750462;source=dbSNP) | 0.001506-0.003704 | | C | Tolerated | Benign | 0/1/331 | 0/5/670 | 0.669 | 0.4 |
| **CD33** | 19:51738917 | c.G910A | p.G304R | [rs35112940](http://browser.1000genomes.org/Homo_sapiens/Variation/Summary?db=core;g=ENSG00000105383;r=19:51728320-51747115;t=ENST00000262262;v=rs35112940;vf=11532305;source=dbSNP) | 0.2009-0.2151 | 0.1603 | A | Tolerated | Benign | 10/113/208 | 27/236/411 | 0.581 | 0.924 |
| **CD33** | 19:51728477 | c.C41T | p.A14V | [rs12459419](http://browser.1000genomes.org/Homo_sapiens/Variation/Summary?db=core;g=ENSG00000105383;r=19:51728320-51747115;t=ENST00000262262;v=rs12459419;vf=8711715;source=dbSNP) | 0.3106-0.3207 | 0.2939 | T | Deleterious | Benign | 29/147/154 | 75/281/316 | 0.946 | 1.014 |
| **CD33** | 19:51728641 | c.A205G | p.R69G | [rs2455069](http://browser.1000genomes.org/Homo_sapiens/Variation/Summary?db=core;g=ENSG00000105383;r=19:51728320-51747115;t=ENST00000262262;v=rs2455069;vf=2042703;source=dbSNP) | 0.4258-0.4302 | 0.3577 | G | Tolerated | Benign | 60/161/109 | 131/317/225 | 0.943 | 1.018 |
| ***CLU*** | 8:27461872 | c.C870G | p.H290Q | NOVEL | 0-0.0007396 |  | G | Deleterious | Possibly damaging | 0/0/332 | 0/1/675 | 1 | 0 |
| ***CLU*** | 8:27466474 | c.A227C | p.E76A | [rs372043736](http://browser.1000genomes.org/Homo_sapiens/Variation/Summary?db=core;g=ENSG00000120885;r=8:27454434-27472548;t=ENST00000316403;v=rs372043736;vf=60501068;source=dbSNP) | 0-0.0007396 | 0.00001647 | C | Deleterious | Benign | 0/0/332 | 0/1/675 | 1 | 0 |
| ***CLU*** | 8:27457512 | c.A949C | p.N317H | [rs9331936](http://browser.1000genomes.org/Homo_sapiens/Variation/Summary?db=core;g=ENSG00000120885;r=8:27454434-27472548;t=ENST00000316403;v=rs9331936;vf=5933842;source=dbSNP) | 0.001506-0 | 0.01922 | C | Deleterious | Probably damaging | 0/1/331 | 0/0/676 | 0.329 | Inf |
| ***CLU*** | 8:27462725 | c.G545A | p.R182H | [rs201670453](http://browser.1000genomes.org/Homo_sapiens/Variation/Summary?db=core;g=ENSG00000120885;r=8:27454434-27472548;t=ENST00000316403;v=rs201670453;vf=55752442;source=dbSNP) | 0.001506-0 | 0.000511 | A | Tolerated | Benign | 0/1/331 | 0/0/676 | 0.329 | Inf |
| ***CLU*** | 8:27462744 | c.A526G | p.M176V | NOVEL | 0.001506-0 |  | G | Deleterious | Benign | 0/1/331 | 0/0/676 | 0.329 | Inf |
| ***CLU*** | 8:27462662 | c.C608T | p.T203I | [rs41276297](http://browser.1000genomes.org/Homo_sapiens/Variation/Summary?db=core;g=ENSG00000120885;r=8:27454434-27472548;t=ENST00000560366;v=rs41276297;vf=12457488;source=dbSNP) | 0.001506-0.003698 | 0.001673 | T | Tolerated | Benign | 0/1/331 | 0/5/671 | 0.669 | 0.4 |
| ***CR1*** | 1:207741336 | c.C2770G | p.P924A | rs760418677 | 0.001506-0 | 0.00007469 | G | Tolerated | Possibly damaging | 0/1/331 | 0/0/676 | 0.329 | Inf |
| ***CR1*** | 1:207782916 | c.A4828T | p.T1610S | [rs4844609](http://browser.1000genomes.org/Homo_sapiens/Variation/Summary?db=core;g=ENSG00000203710;r=1:207669492-207813992;t=ENST00000400960;v=rs4844609;vf=3721394;source=dbSNP) | 0.03313-0.02515 | 0.0147 | T | Tolerated | Benign | 1/20/311 | 0/34/642 | 0.460 | 1.27 |
| ***CR1*** | 1:207679348 | c.G221A | p.R74H | [rs200913967](http://browser.1000genomes.org/Homo_sapiens/Variation/Summary?db=core;g=ENSG00000203710;r=1:207669492-207813992;t=ENST00000400960;v=rs200913967;vf=54950577;source=dbSNP) | 0.001506-0.002219 | 0.0008698 | A | Tolerated | Benign | 0/1/331 | 0/3/673 | 1 | 0.677 |
| ***CR1*** | 1:207680071 | c.G314A | p.R105H | [rs56102840](http://browser.1000genomes.org/Homo_sapiens/Variation/Summary?db=core;g=ENSG00000203710;r=1:207669492-207813992;t=ENST00000400960;v=rs56102840;vf=12900432;source=dbSNP) | 0-0.0007396 | 0.00004973 | A | Tolerated | Benign | 0/0/332 | 0/1/675 | 1 | 0 |
| ***CR1*** | 1:207680127 | c.G370A | p.G124R | rs55962594 | 0-0.0007396 | 0.00005806 | A | Tolerated | Possibly damaging | 0/0/332 | 0/1/675 | 1 | 0 |
| ***CR1*** | 1:207680154 | c.A397G | p.K133E | [rs183171969](http://browser.1000genomes.org/Homo_sapiens/Variation/Summary?db=core;g=ENSG00000203710;r=1:207669492-207813992;t=ENST00000400960;v=rs183171969;vf=43480330;source=dbSNP) | 0-0.0007396 | 0.0002253 | G | Tolerated | Benign | 0/0/332 | 0/1/675 | 1 | 0 |
| ***CR1*** | 1:207739203 | c.C2537T | p.S846F | [rs199990810](http://browser.1000genomes.org/Homo_sapiens/Variation/Summary?db=core;g=ENSG00000203710;r=1:207669492-207813992;t=ENST00000400960;v=rs199990810;vf=54082263;source=dbSNP) | 0-0.0007396 | 0.000365 | T | Tolerated | Possibly damaging | 0/0/332 | 0/1/675 | 1 | 0 |
| ***CR1*** | 1:207741193 | c.T2627C | p.V876A | [rs149099494](http://browser.1000genomes.org/Homo_sapiens/Variation/Summary?db=core;g=ENSG00000203710;r=1:207669492-207813992;t=ENST00000400960;v=rs149099494;vf=39179268;source=dbSNP) | 0-0.0007396 | 0.00595 | C | Tolerated | Benign | 0/0/332 | 0/1/675 | 1 | 0 |
| ***CR1*** | 1:207751179 | c.G3217A | p.A1073T | [rs187750583](http://browser.1000genomes.org/Homo_sapiens/Variation/Summary?db=core;g=ENSG00000203710;r=1:207669492-207813992;t=ENST00000400960;v=rs187750583;vf=48064416;source=dbSNP) | 0-0.0007396 | 0.0004809 | A | Tolerated | Possibly damaging | 0/0/332 | 0/1/675 | 1 | 0 |
| ***CR1*** | 1:207751252 | c.T3290C | p.L1097P | [rs200111726](http://browser.1000genomes.org/Homo_sapiens/Variation/Summary?db=core;g=ENSG00000203710;r=1:207669492-207813992;t=ENST00000400960;v=rs200111726;vf=54197937;source=dbSNP) | 0-0.0007407 | 0.003333 | C | Tolerated | Possibly damaging | 0/0/332 | 0/1/674 | 1 | 0 |
| ***CR1*** | 1:207751260 | c.A3298G | p.R1100G | [rs202070239](http://browser.1000genomes.org/Homo_sapiens/Variation/Summary?db=core;g=ENSG00000203710;r=1:207669492-207813992;t=ENST00000400960;v=rs202070239;vf=56112393;source=dbSNP) | 0-0.0007407 | 0.003284 | G | Tolerated | Benign | 0/0/332 | 0/1/674 | 1 | 0 |
| ***CR1*** | 1:207760830 | c.G4280A | p.R1427H | [rs373049995](http://browser.1000genomes.org/Homo_sapiens/Variation/Summary?db=core;g=ENSG00000203710;r=1:207669492-207813992;t=ENST00000400960;v=rs373049995;vf=61405421;source=dbSNP) | 0-0.0007396 | 0.0001491 | A | Tolerated | Benign | 0/0/332 | 0/1/675 | 1 | 0 |
| ***CR1*** | 1:207760852 | c.G4302A | p.M1434I | [rs140566582](http://browser.1000genomes.org/Homo_sapiens/Variation/Summary?db=core;g=ENSG00000203710;r=1:207669492-207813992;t=ENST00000400960;v=rs140566582;vf=31923289;source=dbSNP) | 0-0.001479 | 0.0006462 | A | Tolerated | Benign | 0/0/332 | 0/2/674 | 1 | 0 |
| ***CR1*** | 1:207785022 | c.T4946C | p.V1649A | rs541247689 | 0-0.0007396 | 0.00004155 | C | Tolerated | Benign | 0/0/332 | 0/1/675 | 1 | 0 |
| ***CR1*** | 1:207785099 | c.G5023T | p.V1675L | [rs202148801](http://browser.1000genomes.org/Homo_sapiens/Variation/Summary?db=core;g=ENSG00000203710;r=1:207669492-207813992;t=ENST00000400960;v=rs202148801;vf=56173860;source=dbSNP) | 0.003012-0.003698 | 0.001747 | T | Tolerated | Benign | 0/2/330 | 0/5/671 | 1 | 0.81 |
| ***CR1*** | 1:207787763 | c.G5240A | p.G1747D | [rs200692346](http://browser.1000genomes.org/Homo_sapiens/Variation/Summary?db=core;g=ENSG00000203710;r=1:207669492-207813992;t=ENST00000400960;v=rs200692346;vf=55075366;source=dbSNP) | 0-0.0007396 | 0.0007873 | A | Deleterious | Benign | 0/0/332 | 0/1/675 | 1 | 0 |
| ***CR1*** | 1:207790017 | c.C5409G | p.C1803W | rs749885738 | 0-0.0007396 | 0.00003312 | G | Deleterious | Probably damaging | 0/0/332 | 0/1/675 | 1 | 0 |
| ***CR1*** | 1:207790110 | c.C5502A | p.S1834R | NOVEL | 0-0.0007396 |  | A | Deleterious | Probably damaging | 0/0/332 | 0/1/675 | 1 | 0 |
| ***CR1*** | 1:207782856 | c.A4768G | p.K1590E | [rs17047660](http://browser.1000genomes.org/Homo_sapiens/Variation/Summary?db=core;g=ENSG00000203710;r=1:207669492-207813992;t=ENST00000400960;v=rs17047660;vf=9767847;source=dbSNP) | 0-0.002219 | 0.02141 | G | Tolerated | Possibly damaging | 0/0/332 | 0/3/673 | 0.554 | 0 |
| ***CR1*** | 1:207782769 | c.G4681A | p.V1561M | [rs41274768](http://browser.1000genomes.org/Homo_sapiens/Variation/Summary?db=core;g=ENSG00000203710;r=1:207669492-207813992;t=ENST00000400960;v=rs41274768;vf=12419488;source=dbSNP) | 0.0256-0.02959 | 0.02487 | A | Tolerated | Possibly damaging | 0/17/315 | 1/38/637 | 0.770 | 0.88 |
| ***CR1*** | 1:207791434 | c.A5558G | p.K1853R | [rs41274770](http://browser.1000genomes.org/Homo_sapiens/Variation/Summary?db=core;g=ENSG00000203710;r=1:207669492-207813992;t=ENST00000400960;v=rs41274770;vf=12419489;source=dbSNP) | 0.03464-0.02441 | 0.0153 | G | Tolerated | Benign | 0/23/309 | 1/31/644 | 0.183 | 1.49 |
| ***CR1*** | 1:207680157 | c.G400A | p.G134R | rs767211812 | 0.003012-0.0007396 | 0.00008366 | A | Deleterious | Probably damaging | 0/2/330 | 0/1/675 | 0.253 | 4.084 |
| ***CR1*** | 1:207680070 | c.C313T | p.R105C | [rs11587944](http://browser.1000genomes.org/Homo_sapiens/Variation/Summary?db=core;g=ENSG00000203710;r=1:207669492-207813992;t=ENST00000400960;v=rs11587944;vf=7851524;source=dbSNP) | 0.009036-0.01479 | 0.007601 | T | Tolerated | Possibly damaging | 0/6/326 | 1/18/657 | 0.395 | 0.636 |
| ***CR1*** | 1:207782889 | c.A4801G | p.R1601G | [rs17047661](http://browser.1000genomes.org/Homo_sapiens/Variation/Summary?db=core;g=ENSG00000203710;r=1:207669492-207813992;t=ENST00000400960;v=rs17047661;vf=9767848;source=dbSNP) | 0.001506-0.004438 | 0.05483 | G | Tolerated | Benign | 0/1/331 | 0/6/670 | 0.436 | 0.33 |
| ***CR1*** | 1:207760772 | c.A4222G | p.T1408A | [rs61734514](http://browser.1000genomes.org/Homo_sapiens/Variation/Summary?db=core;g=ENSG00000203710;r=1:207669492-207813992;t=ENST00000400960;v=rs61734514;vf=14589815;source=dbSNP) | 0.03464-0.03107 | 0.01843 | G | Tolerated | Benign | 1/21/310 | 1/40/635 | 0.782 | 1.099 |
| **CR1** | 1:207782707 | c.A4619G | p.N1540S | [rs17259045](http://browser.1000genomes.org/Homo_sapiens/Variation/Summary?db=core;g=ENSG00000203710;r=1:207669492-207813992;t=ENST00000400960;v=rs17259045;vf=9885560;source=dbSNP) | 0.1099-0.1095 | 0.08897 | G | Tolerated | Benign | 4/65/263 | 8/132/536 | 1 | 1 |
| **CR1** | 1:207795320 | c.A5905G | p.T1969A | [rs2296160](http://browser.1000genomes.org/Homo_sapiens/Variation/Summary?db=core;g=ENSG00000203710;r=1:207669492-207813992;t=ENST00000400960;v=rs2296160;vf=1873089;source=dbSNP) | 0.2078-0.1711 | 0.1841 | G | Tolerated | Benign | 22/94/216 | 17/197/461 | 0.317 | 1.15 |
| **CR1** | 1:207782931 | c.A4843G | p.I1615V | [rs6691117](http://browser.1000genomes.org/Homo_sapiens/Variation/Summary?db=core;g=ENSG00000203710;r=1:207669492-207813992;t=ENST00000400960;v=rs6691117;vf=4528075;source=dbSNP) | 0.2274-0.2101 | 0.3341 | G | Tolerated | Benign | 17/117/198 | 33/218/425 | 0.334 | 1.14 |
| **CR1** | 1:207760773 | c.C4223T | p.T1408M | [rs3737002](http://browser.1000genomes.org/Homo_sapiens/Variation/Summary?db=core;g=ENSG00000203710;r=1:207669492-207813992;t=ENST00000400960;v=rs3737002;vf=2812291;source=dbSNP) | 0.2831-0.2911 | 0.275 | T | Tolerated | Possibly damaging | 26/136/170 | 54/285/336 | 0.687 | 0.944 |
| **CR1** | 1:207790088 | c.C5480G | p.P1827R | rs3811381 | 0.1955-0.1748 | 0.2403 | G | Tolerated | Benign | 13/103/214 | 24/188/463 | 0.251 | 1.18 |
| **CR1** | 1:207753621 | c.A3623G | p.H1208R | [rs2274567](http://browser.1000genomes.org/Homo_sapiens/Variation/Summary?db=core;g=ENSG00000203710;r=1:207669492-207813992;t=ENST00000400960;v=rs2274567;vf=1851885;source=dbSNP) | 0.1973-0.1778 | 0.251 | G | Tolerated | Possibly damaging | 13/105/214 | 26/187/459 | 0.225 | 1.18 |
| ***EPHA1*** | 7:143095907 | c.G1123A | p.G375S | [rs149370167](http://browser.1000genomes.org/Homo_sapiens/Variation/Summary?db=core;g=ENSG00000146904;r=7:143087382-143105985;t=ENST00000275815;v=rs149370167;vf=39362729;source=dbSNP) | 0.001506-0 | 0.0001187 | A | Tolerated | Benign | 0/1/331 | 0/0/676 | 0.329 | Inf |
| ***EPHA1*** | 7:143092269 | c.C2090T | p.P697L | rs34372369 | 0.04669-0.04882 | 0.04929 | T | Tolerated | Possibly damaging | 2/27/303 | 3/60/613 | 0.816 | 0.931 |
| ***EPHA1*** | 7:143088576 | c.T2905C | p.C969R | [rs61732993](http://browser.1000genomes.org/Homo_sapiens/Variation/Summary?db=core;g=ENSG00000146904;r=7:143087382-143105985;t=ENST00000275815;v=rs61732993;vf=14367601;source=dbSNP) | 0.001506-0.002219 | 0.0003652 | C | Tolerated | Possibly damaging | 0/1/331 | 0/3/673 | 1 | 0.677 |
| ***EPHA1*** | 7:143088779 | c.G2786A | p.R929H | [rs201365734](http://browser.1000genomes.org/Homo_sapiens/Variation/Summary?db=core;g=ENSG00000146904;r=7:143087382-143105985;t=ENST00000275815;v=rs201365734;vf=55378648;source=dbSNP) | 0-0.0007396 | 0.0002313 | A | Tolerated | Possibly damaging | 0/0/332 | 0/1/675 | 1 | 0 |
| ***EPHA1*** | 7:143095849 | c.C1181T | p.P394L | [rs140236236](http://browser.1000genomes.org/Homo_sapiens/Variation/Summary?db=core;g=ENSG00000146904;r=7:143087382-143105985;t=ENST00000275815;v=rs140236236;vf=31631945;source=dbSNP) | 0-0.0007396 | 0.0003166 | T | Deleterious | Probably damaging | 0/0/332 | 0/1/675 | 1 | 0 |
| ***EPHA1*** | 7:143095979 | c.C1051T | p.R351C | [rs56006153](http://browser.1000genomes.org/Homo_sapiens/Variation/Summary?db=core;g=ENSG00000146904;r=7:143087382-143105985;t=ENST00000275815;v=rs56006153;vf=12851781;source=dbSNP) | 0-0.0007396 | 0.0001114 | T | Deleterious | Benign | 0/0/332 | 0/1/675 | 1 | 0 |
| ***EPHA1*** | 7:143096369 | c.C973T | p.P325S | NOVEL | 0-0.0007396 |  | T | Tolerated | Benign | 0/0/332 | 0/1/675 | 1 | 0 |
| ***EPHA1*** | 7:143096809 | c.T770G | p.V257G | [rs201380861](http://browser.1000genomes.org/Homo_sapiens/Variation/Summary?db=core;g=ENSG00000146904;r=7:143087382-143105985;t=ENST00000275815;v=rs201380861;vf=55511732;source=dbSNP) | 0-0.0007418 |  | G | Deleterious | Possibly damaging | 0/0/329 | 0/1/673 | 1 | 0 |
| ***EPHA1*** | 7:143098598 | c.A251G | p.N84S | [rs142191815](http://browser.1000genomes.org/Homo_sapiens/Variation/Summary?db=core;g=ENSG00000146904;r=7:143087382-143105985;t=ENST00000275815;v=rs142191815;vf=33230180;source=dbSNP) | 0-0.0007396 | 0.0002481 | G | Tolerated | Benign | 0/0/332 | 0/1/675 | 1 | 0 |
| ***EPHA1*** | 7:143098605 | c.C244T | p.R82C | [rs74721927](http://browser.1000genomes.org/Homo_sapiens/Variation/Summary?db=core;g=ENSG00000146904;r=7:143087382-143105985;t=ENST00000275815;v=rs74721927;vf=18378688;source=dbSNP) | 0-0.0007396 | 0.00002481 | T | Deleterious | Possibly damaging | 0/0/332 | 0/1/675 | 1 | 0 |
| ***EPHA1*** | 7:143105828 | c.G71A | p.R24H | [rs79587607](http://browser.1000genomes.org/Homo_sapiens/Variation/Summary?db=core;g=ENSG00000146904;r=7:143087382-143105985;t=ENST00000275815;v=rs79587607;vf=22981242;source=dbSNP) | 0.003012-0.003704 | | A | Tolerated | Benign | 0/2/330 | 1/3/671 | 1 | 1.016 |
| ***EPHA1*** | 7:143088584 | c.G2897A | p.R966H | [rs139482378](http://browser.1000genomes.org/Homo_sapiens/Variation/Summary?db=core;g=ENSG00000146904;r=7:143087382-143105985;t=ENST00000275815;v=rs139482378;vf=30924740;source=dbSNP) | 0.001506-0.0007396 | 0.0006136 | A | Deleterious | Probably damaging | 0/1/331 | 0/1/675 | 0.550 | 2.037 |
| ***EPHA1*** | 7:143095153 | c.G1475A | p.R492Q | [rs11768549](http://browser.1000genomes.org/Homo_sapiens/Variation/Summary?db=core;g=ENSG00000146904;r=7:143087382-143105985;t=ENST00000275815;v=rs11768549;vf=8040679;source=dbSNP) | 0.0256-0.01479 | 0.0121 | A | Tolerated | Benign | 0/17/315 | 1/18/657 | 0.071 | 1.864 |
| ***EPHA1*** | 7:143096020 | c.G1010A | p.R337Q | [rs201581948](http://browser.1000genomes.org/Homo_sapiens/Variation/Summary?db=core;g=ENSG00000146904;r=7:143087382-143105985;t=ENST00000275815;v=rs201581948;vf=55590404;source=dbSNP) | 0.001506-0 | 0.000357 | A | Tolerated | Benign | 0/1/331 | 0/0/676 | 0.329 | Inf |
| **EPHA1** | 7:143088867 | c.A2698G | p.M900V | [rs6967117](http://browser.1000genomes.org/Homo_sapiens/Variation/Summary?db=core;g=ENSG00000146904;r=7:143087382-143105985;t=ENST00000275815;v=rs6967117;vf=4793801;source=dbSNP) | 0.06949-0.0638 | 0.0634 | C | Tolerated | Benign | 4/38/289 | 3/80/591 | 0.919 | 1.031 |
| **EPHA1** | 7:143097100 | c.T479C | p.V160A | [rs149370167](http://browser.1000genomes.org/Homo_sapiens/Variation/Summary?db=core;g=ENSG00000146904;r=7:143087382-143105985;t=ENST00000275815;v=rs149370167;vf=39362729;source=dbSNP) | 0.07553-0.06899 | | G | Tolerated | Benign | 3/44/284 | 2/89/583 | 0.770 | 1.6 |
| ***MS4A6A*** | 11:59949058 | c.T143C | p.I48T | [rs61742546](http://browser.1000genomes.org/Homo_sapiens/Variation/Summary?db=core;g=ENSG00000110077;r=11:59939081-59952139;t=ENST00000323961;v=rs61742546;vf=14341601;source=dbSNP) | 0.02711-0.02885 | 0.01537 | C | Deleterious | Possibly damaging | 0/18/314 | 0/39/637 | 0.885 | 0.936 |
| **MS4A6A** | 11:59940599 | c.A553T | p.T185S | [rs7232](http://browser.1000genomes.org/Homo_sapiens/Variation/Summary?db=core;g=ENSG00000110077;r=11:59939081-59952139;t=ENST00000323961;v=rs7232;vf=35829;source=dbSNP) | 0.3313-0.3741 | 0.3131 | A | Deleterious | Benign | 36/148/148 | 103/299/273 | 0.221 | 0.844 |
| ***MS4A6A*** | 11:59940532 | c.G620A | p.R207Q | rs146398167 | 0.001506-0 | 0.00004118 | A | Tolerated | Benign | 0/1/331 | 0/0/676 | 0.329 | Inf |
| ***PICALM*** | 11:85685839 | c.G1835T | p.S612I | rs370710573 | 0-0.0007396 | 0.00004119 | T | Deleterious | Benign | 0/0/332 | 0/1/675 | 1 | 0 |
| ***PICALM*** | 11:85687719 | c.C1765G | p.P589A | [rs147556602](http://browser.1000genomes.org/Homo_sapiens/Variation/Summary?db=core;g=ENSG00000073921;r=11:85668727-85780924;t=ENST00000526033;v=rs147556602;vf=37751333;source=dbSNP) | 0-0.0007396 | 0.0002729 | G | Tolerated | Benign | 0/0/332 | 0/1/675 | 1 | 0 |
| ***PICALM*** | 11:85707896 | c.G1231C | p.A411P | [rs34013602](http://browser.1000genomes.org/Homo_sapiens/Variation/Summary?db=core;g=ENSG00000073921;r=11:85668727-85780924;t=ENST00000526033;v=rs34013602;vf=10469567;source=dbSNP) | 0.001506-0.002219 | 0.001533 | C | Tolerated | Benign | 0/1/331 | 0/3/673 | 1 | 0.677 |
| ***PICALM*** | 11:85707933 | c.G1194T | p.Q398H | rs372958249 | 0.001506-0 | 0.000008246 | T |  |  | 0/1/331 | 0/0/676 | 0.329 | Inf |
| ***PICALM*** | 11:85701307 | c.A1373G | p.H458R | rs117411388 | 0.001506-0.0007396 | 0.0007449 | G | Tolerated | Benign | 0/1/331 | 0/1/675 | 0.550 | 2.037 |
| ***PICALM*** | 11:85779721 | c.C102G | p.I34M | [rs146840505](http://browser.1000genomes.org/Homo_sapiens/Variation/Summary?db=core;g=ENSG00000073921;r=11:85668727-85780924;t=ENST00000526033;v=rs146840505;vf=37142546;source=dbSNP) | 0.00303-0.002219 | 0.001638 | G | Tolerated | Benign | 0/2/328 | 0/3/673 | 0.665 | 1.36 |

**Table S1. Common, low frequency and rare coding variants detected in our cohort**. Position is in hg19/GRCh37. MAF, minor allele frequency; OR, odds ratio; Inf, infinity. Highlighted in grey and blue, singletons and common coding variants, respectively. These variants have been excluded from the study. If we would have included also the common coding variants (16 variants) in the single-variants based analysis, the p-value for the statistical significance would have been p<8.9x10^-4^ (0.05/56 coding variants).

| **GENE** | **CHR** | **BP1** | **BP2** | **KB** | **N SNPS** | **SNPS** |
| --- | --- | --- | --- | --- | --- | --- |
| ***CR1*** | 1 | 207753621 | 207795320 | 41.7 | 6 | 1:207753621\|1:207760773\|1:207782707\|1:207782931\|1:207790088\|1:207795320 |
| ***CR1*** | 1 | 207812791 | 207813601 | 0.811 | 3 | 1:207812791\|1:207813556\|1:207813601 |
| ***CR1*** | 1 | 207814451 | 207872595 | 58.145 | 7 | 1:207814451\|1:207814835\|1:207850879\|1:207851554\|1:207851611\|1:207857254\|1:207872595 |
| ***BIN1*** | 2 | 127816632 | 127826533 | 9.902 | 2 | 2:127816632\|2:127826533 |
| ***CD2A6*** | 6 | 47594002 | 47594722 | 0.721 | 2 | 6:47594002\|6:47594722 |
| ***EPHA1*** | 7 | 143088085 | 143088526 | 0.442 | 2 | 7:143088085\|7:143088526 |
| ***EPHA1*** | 7 | 143088823 | 143088867 | 0.045 | 2 | 7:143088823\|7:143088867 |
| ***CLU*** | 8 | 27454682 | 27455442 | 0.761 | 2 | 8:27454682\|8:27455442 |
| ***CLU*** | 8 | 27462481 | 27468862 | 6.382 | 2 | 8:27462481\|8:27468862 |
| ***MS4A6A*** | 11 | 59940599 | 59945745 | 5.147 | 2 | 11:59940599\|11:59945745 |
| ***PICALM*** | 11 | 85627108 | 85630837 | 3.73 | 3 | 11:85627108\|11:85630411\|11:85630837 |
| ***ABCA7*** | 19 | 1038871 | 1042809 | 3.939 | 5 | 19:1038871\|19:1038893\|19:1038995\|19:1041352\|19:1042809 |
| ***ABCA7*** | 19 | 1051214 | 1052005 | 0.792 | 2 | 19:1051214\|19:1052005 |
| ***ABCA7*** | 19 | 1054060 | 1056065 | 2.006 | 3 | 19:1054060\|19:1055191**\|19:1056065 |
| ***ABCA7*** | 19 | 1056492 | 1068738 | 12.247 | 7 | 19:1056492*****\|19:1064193\|19:1065018\|19:1065044\|19:1065563\|19:1068734\|19:1068738 |
| ***CD33*** | 19 | 51728477 | 51738917 | 10.441 | 3 | 19:51728477\|19:51728641\|19:51738917 |

**Table S2**. **Common haplotype blocks (Minor allele frequency >0.05) identified in our cohort**. CHR, chromosome; BP, base pair; KB, kilobases; N, number; SNPS, single nucleotide polymorphisms. Position is in hg19/GRCh37.*GWAS hit (rs3752246). **Common coding variant (rs3745842), nominal significant in our cohort.

|  | **Variants in the main GWAS genes (NIH-UCL series)** | | | | | | | | |
| --- | --- | --- | --- | --- | --- | --- | --- | --- | --- |
| **CTRLS ID** | ***ABCA7*** | ***BIN1*** | ***CD2AP*** | ***CD33*** | ***CLU*** | ***CR1*** | ***EPHA1*** | ***MS4A6A*** | ***PICALM*** |
| **019_11** | G215S |  |  |  |  | T1408A |  |  |  |
| **033-11** | G215S, R810G |  |  |  |  |  |  |  |  |
| **92-32** |  |  |  |  |  | R105H, T1408A |  |  |  |
| **97-45** | G215S, A877T |  |  |  |  | R74H |  | I48T |  |
| **C04_99** | Q1686R |  |  | S305P |  |  |  |  |  |
| **C07_00** | G215S |  |  |  |  |  |  | I48T |  |
| **C07_97** |  | P183S |  |  |  | T1408A |  |  |  |
| **C09_97** |  |  |  |  |  | K1853R |  | I48T |  |
| **C15_94** |  |  |  |  |  | T1408A | P697L |  |  |
| **C19_93** | G215S | K542R | L331H |  |  |  |  |  |  |
| **C33_93** | G215S |  |  |  |  | T1408A |  |  |  |
| **N168** | G215S, V1599M |  |  |  |  |  |  |  |  |
| **N176** | Q1686R |  |  |  |  |  | P697L |  |  |
| **N181** |  |  |  |  |  | V1561M |  | I48T | S612I |
| **N183** |  |  |  |  |  | R105H, V1561M |  |  |  |
| **N188** |  |  |  |  |  | K1853R, | R492Q |  |  |
| **P23_07** |  |  |  |  |  | T1408A | P697L |  |  |
| **SH-01-31** | N718T, Q1686R |  |  |  | Q1686R |  |  |  |  |
| **SH-02-12** | Q1686R |  |  |  |  | K1853R |  |  |  |
| **SH-04-19** | N718T, Q1686R |  |  |  |  |  |  |  |  |
| **SH-06-05** |  |  |  |  |  | V1561M | P697L |  |  |
| **UMARY-5088** | G215S |  |  |  |  |  | P697L |  |  |

**Table S3a. Cases carrying multiple low-frequency and rare variants in the GWAS loci studied, in the NIH-UCL cohort.**

|  | **Variants in the main GWAS genes (NIH-UCL series)** | | | | | | | | |
| --- | --- | --- | --- | --- | --- | --- | --- | --- | --- |
| **CTRLS ID** | ***ABCA7*** | ***BIN1*** | ***CD2AP*** | ***CD33*** | ***CLU*** | ***CR1*** | ***EPHA1*** | ***MS4A6A*** | ***PICALM*** |
| **019_11** | G215S |  |  |  |  | T1408A |  |  |  |
| **033-11** | G215S, R810G |  |  |  |  |  |  |  |  |
| **92-32** |  |  |  |  |  | R105H, T1408A |  |  |  |
| **97-45** | G215S, A877T |  |  |  |  | R74H |  | I48T |  |
| **C04_99** | Q1686R |  |  | S305P |  |  |  |  |  |
| **C07_00** | G215S |  |  |  |  |  |  | I48T |  |
| **C07_97** |  | P183S |  |  |  | T1408A |  |  |  |
| **C09_97** |  |  |  |  |  | K1853R |  | I48T |  |
| **C15_94** |  |  |  |  |  | T1408A | P697L |  |  |
| **C19_93** | G215S | K542R | L331H |  |  |  |  |  |  |
| **C33_93** | G215S |  |  |  |  | T1408A |  |  |  |
| **N168** | G215S, V1599M |  |  |  |  |  |  |  |  |
| **N176** | Q1686R |  |  |  |  |  | P697L |  |  |
| **N181** |  |  |  |  |  | V1561M |  | I48T | S612I |
| **N183** |  |  |  |  |  | R105H, V1561M |  |  |  |
| **N188** |  |  |  |  |  | K1853R | R492Q |  |  |
| **P23_07** |  |  |  |  |  | T1408A | P697L |  |  |
| **SH-01-31** | N718T, Q1686R |  |  |  | Q1686R |  |  |  |  |
| **SH-02-12** | Q1686R |  |  |  |  | K1853R |  |  |  |
| **SH-04-19** | N718T, Q1686R |  |  |  |  |  |  |  |  |
| **SH-06-05** |  |  |  |  |  | V1561M | P697L |  |  |
| **UMARY-5088** | G215S |  |  |  |  |  | P697L |  |  |

**Table S3b. Controls carrying multiple low-frequency and rare variants in the GWAS loci studied, in the NIH-UCL cohort.**

| **GENE** | **TRANSCRIPT** | **TOTAL VARIANT**  **IN OUR COHORT** | **RELATIVE FREQ.**  **OF TOTAL VARIANTS**  **IN OUR COHORT** | **TOTAL VARIANT IN EVS**  **IN European-American** | **RELATIVE FREQ.**  **OF TOTAL VARIANTS IN EVS**  **IN European-American** |
| --- | --- | --- | --- | --- | --- |
| ***ABCA7*** | NM_019112 | 72/1008 | 0.106 | 369/4300 | 0.085 |
| ***CD2AP*** | NM_012120 | 20/1008 | 0.029 | 87/4300 | 0.020 |
| ***MS4A6A*** | NM_152851 | 11/1008 | 0.016 | 41/4300 | 0.0095 |
| ***CR1*** | NM_000651 | 72/1008 | 0.106 | 170/4300 | 0.039 |
| ***BIN1*** | NM_139343 | 27/1008 | 0.026 | 117/4300 | 0.027 |
| ***PICALM*** | NM_007166 | 19/1008 | 0.018 | 106/4300 | 0.024 |
| ***EPHA1*** | NM_005232 | 30/1008 | 0.029 | 167/4300 | 0.038 |
| ***CLU*** | NM_001831 | 29/1008 | 0.028 | 75/4300 | 0.017 |
| ***CD33*** | NM_001772 | 13/1008 | 0.012 | 63/4300 | 0.014 |

**Table S4**. **Relative frequency of total variants in the 9 AD GWAS loci in our cohort and EVS**. Freq., frequency; EVS, exome variant server.

| **Gene** | **Transcript** | **Kbps**  **of coding sequence** | **Relative freq coding variants (coding variants/kbps of coding sequence)** | **Relative freq rare low freq coding variants (low frequency and rare coding variants/kbps of coding sequence)** | **relative frequency of damaging variants (damaging variants/kbps of coding sequence)** |
| --- | --- | --- | --- | --- | --- |
| ***ABCA7*** | NM_019112 | 6.441 | 6.21 (40/6.441) | 5.27 (34/6.44) | 3.72 (24/6.44) |
| ***CD2AP*** | NM_012120 | 1.920 | 3.64 (7/1.92) | 3.64 (7/1.92) | 2.08 (4/1.92) |
| ***MS4A6A*** | NM_152851 | 0.538 | 5.57 (3/0.538) | 3.71 (2/0.538) | 1.85 (1/0.538) |
| ***CR1*** | NM_000651 | 7.470 | 4.14 (31/7.47) | 3.21 (24/7.47) | 1.87 (14/7.47) |
| ***BIN1*** | NM_139343 | 1.783 | 4.48 (8/1.78) | 4.49 (8/1.783) | 3.92 (7/1.783) |
| ***PICALM*** | NM_007166 | 1.960 | 3.06 (6/1.96) | 3.06 (6/1.96) | 0.51 (1/1.96) |
| ***EPHA1*** | NM_005232 | 2.932 | 5.45 (16/2.93) | 5.11 (15/2.932) | 2.72 (8/2.93) |
| ***CLU*** | NM_001831 | 1.350 | 4.44 (6/1.35) | 4.44 (6/1.35) | 2.96 (4/1.35) |
| ***CD33*** | NM_001772 | 1.093 | 9.14 (10/1.093) | 7.31 (8/1.093) | 0.91 (1/1.093) |

**Table S5. Relative frequency of coding and damaging variants in the 9 AD GWAS loci in our cohort.** Freq., frequency; kbps, kilobase pairs.

| **Position** | **MA** | **RS ID** | **Aa change** | **aa/Aa/AA cases** | **aa/Aa/AA controls** | **ExAC MAF** | **P-Value*** | **OR** |
| --- | --- | --- | --- | --- | --- | --- | --- | --- |
| 19:1047002 | G | rs3752234 | p.A608A | 57/95/163 | 148/254/264 | 0.5547 | 0.0004289 | 0.612 |
| 19:1055191 | A | rs3745842 | p.R1349Q | 38/111/164 | 119/261/268 | 0.4433 | 0.001448 | 0.641 |
| 19:1061804 | C | rs78320196 | p.N1829N | 0/17/315 | 1/65/608 | 0.05847 | 0.01049 | 0.497 |
| 19:1053524 | G | rs3752241 | p.L1139L | 5/71/253 | 12/192/467 | 0.2525 | 0.01647 | 0.687 |
| 19:1041347 | C | [rs182233998](http://exac.broadinstitute.org/variant/19-1041347-T-C) | c.7-7T>C | 0/5/327 | 0/30/646 | 0.01511 | 0.0166 | 0.329 |
| 19:1043103 | A | rs72973581 | p.G215S | 0/31/301 | 1/96/579 | 0.04316 | 0.02665 | 0.615 |
| 19:1065044 | T | rs4147935 | p.G2053G | 30/102/193 | 72/244/349 | 0.4049 | 0.04164 | 0.755 |
| 19:1050996 | A | rs74176364 | p.A877T | 0/2/330 | 0/16/660 | 0.01692 | 0.07264 | 0.25 |
| 19:1059056 | A | rs114782266 | p.R1812H | 0/10/322 | 0/11/665 | 0.01057 | 0.1625 | 1.87 |
| 19:1064193 | G | rs4147930 | p.L1995L | 19/115/191 | 34/275/363 | 0.7303 | 0.1739 | 0.824 |
| 19:1041352 | G | rs3752229 | c.-7-2A>G | 2/36/294 | 1/59/616 | 0.06973 | 0.2136 | 1.326 |
| 19:1057343 | A | rs117187003 | p.V1599M | 0/4/328 | 0/3/673 | 0.003085 | 0.2269 | 2.73 |
| 19:1041909 | T | rs144546979 | p.T80T | 0/2/330 | 0/1/675 | 0.000144 | 0.2538 | 4.08 |
| 19:1052086 | A | rs61576791 | p.T1036T | 0/2/330 | 0/1/675 | 0.01736 | 0.2538 | 4.084 |
| 19:1065563 | G | rs2242437 | 3'UTR | 18/119/190 | 36/270/363 | 0.395 | 0.2774 | 0.855 |
| 19:1047537 | C | rs3752239 | p.N718T | 0/11/321 | 0/33/641 | 0.07028 | 0.3251 | 0.665 |
| 19:1043794 | A | rs147846250 | p.R334Q | 0/1/331 | 0/0/676 | 0.0002 | 0.329 | Inf |
| 19:1044619 | G | rs146982710 | p.P364R | 0/1/331 | 0/0/676 | 0.000384 | 0.329 | Inf |
| 19:1046239 | G | rs141428162 | p.P486A | 0/1/331 | 0/0/676 | 0.000321 | 0.3294 | Inf |
| 19:1047372 | A | NOVEL | p.A688T | 0/1/331 | 0/0/676 | NA | 0.3294 | Inf |
| 19:1053521 | G | NOVEL | p.S1138R | 0/1/331 | 0/0/676 | 2.87E-05 | 0.3294 | Inf |
| 19:1056372 | G | NA | p.V1487G | 0/1/331 | 0/0/676 | 0.000125 | 0.3294 | inf |
| 19:1056941 | A | rs145632609 | p.C1541Y | 0/1/331 | 0/0/676 | 4.13E-05 | 0.3294 | Inf |
| 19:1056958 | C | NA | p.S1547P | 0/1/331 | 0/0/676 | 8.26E-06 | 0.3294 | Inf |
| 19:1057366 | T | NOVEL | p.Q1606H | 0/1/331 | 0/0/676 | NA | 0.3294 | Inf |
| 19:1058655 | A | NOVEL | p.V1730I | 0/1/331 | 0/0/676 | NA | 0.3294 | inf |
| 19:1044712 | G | rs3764647 | p.H395R | 0/17/315 | 0/45/630 | 0.06298 | 0.4032 | 0.755 |
| 19:1043748 | G | rs3752232 | p.T319A | 0/17/315 | 0/44/630 | 0.06039 | 0.4035 | 0.77 |
| 19:1058176 | G | rs4147918 | p.Q1686R | 0/17/315 | 0/43/633 | 0.04785 | 0.4812 | 0.794 |
| 19:1049269 | A | rs4147914 | p.L795L | 6/65/255 | 12/147/511 | 0.1781 | 0.5221 | 0.894 |
| 19:1056109 | T | rs145232000 | p.S1428L | 0/1/331 | 0/1/675 | 0.000199 | 0.5505 | 2.037 |
| 19:1056918 | T | NA | p.S1533S | 0/1/331 | 0/1/675 | 0.000925 | 0.5505 | 2.037 |
| 19:1053382 | G | NA | p.V1092G | 1/0/329 | 0/1/670 | 0.000806 | 0.5509 | 2.034 |
| 19:1062192 | C | rs4147921 | A1864A | 0/16/315 | 0/40/635 | 0.04819 | 0.5592 | 0.8 |
| 19:1043747 | T | rs149023827 | p.L318L | 0/3/329 | 0/10/665 | 0.001779 | 0.5623 | 0.6 |
| 19:1065018 | T | rs4147934 | p.A2045S | 114/70/139 | 179/179/287 | 0.7317 | 0.6808 | 1.061 |
| 19:1041852 | T | rs3764644 | p.L61L | 0/16/315 | 0/36/631 | 0.05701 | 0.7641 | 0.89 |
| 19:1042809 | G | rs3764645 | p.E188G | 67/165/99 | 154/327/195 | 0.4838 | 0.768 | 0.9500902 |
| 19:1051214 | G | rs3752240 | p.V915V | 42/157/132 | 86/313/275 | 0.3361 | 0.785 | 1.039 |
| 19:1056492 | C | rs3752246 | p.G1527A | 13/91/227 | 13/193/466 | 0.16 | 0.8276 | 1.036 |
| 19:1052005 | T | rs3764652 | p.A1009A | 61/162/104 | 130/330/210 | 0.4256 | 0.8848 | 0.978 |
| 19:1041289 | T | NOVEL | c.231-12C>A | 0/0/332 | 0/1/675 | NA | 1 | 0 |
| 19:1041922 | A | NOVEL | p.L85M | 0/0/332 | 0/1/675 | 0.000257 | 1 | 0 |
| 19:1041950 | C | NOVEL | p.L94P | 0/0/332 | 0/1/675 | NA | 1 | 0 |
| 19:1041951 | A | NOVEL | p.L94L | 0/0/332 | 0/1/675 | NA | 1 | 0 |
| 19:1041971 | G | NA | p.L101R | 0/1/331 | 0/2/674 | 0.000685 | 1 | 1.018 |
| 19:1043350 | T | NA | p.L270L | 0/0/332 | 0/1/675 | 0.000906 | 1 | 0 |
| 19:1044692 | T | NA | p.D388D | 0/0/332 | 0/1/675 | 5.28E-05 | 1 | 0 |
| 19:1044708 | C | NA | p.G394R | 0/0/332 | 0/1/675 | 9.18E-06 | 1 | 0 |
| 19:1046944 | G | rs144979723 | p.A589G | 0/0/332 | 0/1/675 | 0.000747 | 1 | 0 |
| 19:1047169 | C | rs144852598 | p.L620P | 0/0/331 | 0/2/674 | 0.000362 | 1 | 0 |
| 19:1047336 | A | rs59851484 | p.A676T | 0/0/332 | 0/1/675 | 0.01294 | 1 | 0 |
| 19:1048982 | T | rs9282560 | p.C786C | 0/1/331 | 0/2/674 | 0.001928 | 1 | 1.018 |
| 19:1049305 | A | rs4147915 | p.V807V | 3/74/248 | 24/133/508 | 0.1792 | 1 | 1 |
| 19:1049312 | G | NA | p.R810C | 0/0/332 | 0/1/675 | 8.44E-06 | 1 | 0 |
| 19:1049318 | A | NOVEL | p.L812M | 0/0/332 | 0/1/675 | NA | 1 | 0 |
| 19:1051944 | A | rs139214131 | p.R989H | 0/0/332 | 0/2/674 | 0.00068 | 1 | 0 |
| 19:1052017 | A | NOVEL | p.G1013G | 0/0/332 | 0/1/675 | 0.000204 | 1 | 0 |
| 19:1054060 | G | rs3752243 | p.L1176L | 54/173/105 | 127/334/212 | 0.4723 | 1 | 0.99 |
| 19:1054791 | G | NOVEL | p.P1288P | 0/0/332 | 0/1/675 | NA | 1 | 0 |
| 19:1055249 | A | NA | p.P1368P | 0/0/332 | 0/1/675 | 8.79E-05 | 1 | 0 |
| 19:1056065 | G | rs881768 | p.R1413R | 54/161/107 | 128/317/221 | 0.4451 | 1 | 0.99 |
| 19:1056110 | A | NA | p.S1428S | 0/0/332 | 0/1/675 | 0.000121 | 1 | 0 |
| 19:1056126 | T | rs137888610 | p.R1434C | 0/0/332 | 0/1/675 | 0.000154 | 1 | 0 |
| 19:1056421 | A | rs113711363 | p.P1503P | 0/0/332 | 0/1/675 | 0.00593 | 1 | 0 |
| 19:1057047 | A | NA | p.P1576P | 0/0/332 | 0/1/675 | 3.34E-05 | 1 | 0 |
| 19:1057056 | G | rs148266574 | p.Y1579X | 0/0/332 | 0/1/675 | 2.52E-05 | 1 | 0 |
| 19:1058635 | T | rs73505232 | p.S1723L | 0/0/332 | 0/1/675 | 0.01161 | 1 | 0 |
| 19:1059029 | A | rs143615723 | p.R1803H | 0/0/332 | 0/1/675 | 0.000168 | 1 | 0 |
| 19:1063574 | C | NOVEL | p.L1915P | 0/0/332 | 0/1/674 | NA | 1 | 0 |
| 19:1063651 | A | rs151130083 | p.V1941I | 0/0/332 | 0/1/675 | 0.000458 | 1 | 0 |
| 19:1063831 | T | NOVEL | p.E1974X | 0/0/332 | 0/1/675 | NA | 1 | 0 |

**Table S6. Collection of all the variants included in the *ABCA7* gene-based analysis.** Position is in hg19/GRCh37. MAF, minor allele frequency; OR, odds ratio; Inf, infinity.*non corrected. In light blue are the nominal significant variants.

| **GENE** | **Position** | **rsID** | **cDNA**  **change** | **PROTEIN**  **VARIATION** | **EFFECT** | **AD**  **CARRIER** | **CONTROL**  **CARRIER** | **MAF**  **cases-controls (%)** | **MAF in EVS**  **European-American (%)** | **MAF in ExAC**  **European-non Finnish (%)** | **P-value** | **OR** |
| --- | --- | --- | --- | --- | --- | --- | --- | --- | --- | --- | --- | --- |
|  |  |  |  |  |  |  |  |  |  |  |  |  |
| ***ABCA7*** | 19:1057056 | [rs148266574](http://grch37.ensembl.org/Homo_sapiens/ZMenu/Variation?v=rs148266574) | c.C4737G | p.Y1579X | stop gained | 0/0/332 | 0/1/675 | 0-0.74 | 0.0116 | 4.592e-7 | 1 | 0 |
| ***ABCA7*** | 19:1063831 | NOVEL | c.G5920T | p.E1974X | stop gained | 0/0/332 | 0/1/675 | 0-0.74 | NA | NA | 1 | 0 |
| ***ABCA7*** | 19:1041347 | [rs182233998](http://exac.broadinstitute.org/variant/19-1041347-T-C) | c.7-7T>C | NA | near splice site | 0/5/327 | 0/30/646 | 0.753-2.21 | 1.61 | 1.67 | 0.0166 | 0.329 |
| ***ABCA7*** | 19:1041352 | rs3752229 | c.7-2 A>G | NA | splice acceptor | 2/36/294 | 1/59/616 | 6-4.51 | 4.732 | 4.472 | 0.2136 | 1.326 |
| ***ABCA7*** | 19:1041289 | NOVEL | c.231-12C>A | NA | near splice site | 0/0/332 | 0/1/675 | 0-0.74 | NA | NA | 1 | 0 |

**Table S7**. **Loss of function mutations in *ABCA7* detected in our discovery set**. Position is in hg19/GRCh37. MAF, minor allele frequency; EVS, exome variant server, OR, odds ratio.

| **GENE** | **POSITION** | **cDNA change** | **Aa change** | **rs ID** | **MAF** | **MA** | **SIFT** | **POLYPHEN** | **aa/Aa/AA cases** | **aa/Aa/AA ctrls** | **P-VALUE** | **OR** |  |
| --- | --- | --- | --- | --- | --- | --- | --- | --- | --- | --- | --- | --- | --- |
|  |  |  |  |  | **cases-ctrls** |  |  |  |  |  |  |  |  |
| ***ABCA7*** | 19:1057366 | c.G4818T | p.Q1606H | NOVEL | 0.001506-0 | T | Deleterious | Probably damaging | 0/1/331 | 0/0/676 | 0,329 | Inf | WES |
| ***ABCA7*** | 19:1058655 | c.G5188A | p.V1730I | NOVEL | 0.001506-0 | A | Tolerated | Benign | 0/1/331 | 0/0/676 | 0,329 | inf | WES |
| ***ABCA7*** | 19:1041950 | c.T281C | p.L94P | NOVEL | 0-0.0007396 | C | Deleterious | Probably damaging | 0/0/332 | 0/1/675 | 1 | 0 | WES |
| ***ABCA7*** | 19:1049318 | c.C2434A | p.L812M | NOVEL | 0-0.0007396 | A | Deleterious | Probably damaging | 0/0/332 | 0/1/675 | 1 | 0 | WES |
| ***ABCA7*** | 19:1063574 | c.T5744C | p.L1915P | NOVEL | 0-0.0007407 | C | Deleterious | Probably damaging | 0/0/332 | 0/1/674 | 1 | 0 | WGS |
| ***ABCA7*** | 19:1063831 | c.G5920T | p.E1974X | NOVEL | 0-0.0007396 | T | NA | NA | 0/0/332 | 0/1/675 | 1 | 0 | WGS |
| ***BIN1*** | 2:127825804 | c.C547T | p.P183S | NOVEL | 0-0.0007396 | T | Deleterious | Probably damaging | 0/0/332 | 0/1/675 | 1 | 0 | WES |
| ***CD2AP*** | 6:47567069 | c.A1307G | p.K436R | NOVEL | 0.001506-0 | G | Deleterious | Probably damaging | 0/1/331 | 0/0/676 | 0,329 | Inf | WES |
| ***CD2AP*** | 6:47512351 | c.A329C | p.K110T | NOVEL | 0-0.0007396 | C | Deleterious | Benign | 0/0/332 | 0/1/675 | 1 | 0 | WES |
| ***CD2AP*** | 6:47548605 | c.A1014G | p.P338P | NOVEL | 0-0.0007396 | G | NA | NA | 0/0/332 | 0/1/675 | 1 | 0 | WES |
| ***CLU*** | 8:27461872 | c.C870G | p.H290Q | NOVEL | 0-0.0007396 | G | Deleterious | Possibly damaging | 0/0/332 | 0/1/675 | 1 | 0 | WES |
| ***CLU*** | 8:27462744 | c.A526G | p.M176V | NOVEL | 0.001506-0 | G | Deleterious | Benign | 0/1/331 | 0/0/676 | 0,329 | Inf | WES |
| ***CR1*** | 1:207790110 | c.C5502A | p.S1834R | NOVEL | 0-0.0007396 | A | Deleterious | Probably damaging | 0/0/332 | 0/1/675 | 1 | 0 | WGS |
| ***EPHA1*** | 7:143096369 | c.C973T | p.P325S | NOVEL | 0-0.0007396 | T | Tolerated | Benign | 0/0/332 | 0/1/675 | 1 | 0 | WGS |

**Table S8. *ABCA7* novel coding variants detected in our cohort.** Position is in hg19/GRCh37. MA, minor allele; WES, whole exome sequencing; WGS; whole genome sequencing; OR, odds ratio; Inf, infinity.

| **GENE** | **Position** | **rsID** | **cDNA**  **change** | **PROTEIN VARIATION** | **EFFECT** | **AD CARRIER** | **CONTROLS CARRIER** | **MAF**  **cases-controls (%)** | **MAF**  **in EVS**  **European-American (%)** | **MAF**  **in ExAC European-non Finnish (%)** | **P-value** | **OR** | **95% CI** | **Comment** |
| --- | --- | --- | --- | --- | --- | --- | --- | --- | --- | --- | --- | --- | --- | --- |
|  |  |  |  |  |  | **(n = 127)** | **(n = 204)** |  |  |  |  |  |  |  |
| ***ABCA7*** | 19:1055907 | NA | c.4208del1 | p.1402delT | frameshift | 1 | 1 | 0.39-0.24 | 0.0969 | 0.163 | 1 | 1.6 | 0.02-126.9 | reported in the Belgian and Islandic cohort but not associated  to LOAD (1) (2) |
| ***ABCA7*** | [19:1057946](http://exac.broadinstitute.org/variant/19-1057945-T-C) | NA | c.4914_4916del3 | p.1638delCTT | inframe deletion | 0 | 1 | 0-0.24 | 0.0242 | 0 | 1 | 0 | 0.00-62.58 |  |
| ***ABCA7*** | 19:1058711 | NA | c.5475del6 | p.1749delCTACTG | frameshift | 0 | 1 | 0-0.24 | NA | NA | 1.00 | 0.00 | 0.00-62.58 |  |

**Table S9**. **Indels detected in *ABCA7* detected in the NIH-UCL cohort**. Position is in hg19/GRCh37. MAF, minor allele frequency; OR, odds ratio; CI, confidential interval; del, deletion; TOT, total.

| **GENE** | **Position** | **rsID** | **cDNA change** | **PROTEIN VARIATION** | Comment |
| --- | --- | --- | --- | --- | --- |
|  |  |  |  |  |  |
| ***ABCA7*** | 19:1058727 | [rs556286113](http://grch37.ensembl.org/Homo_sapiens/ZMenu/Variation?v=rs556286113) | c.C5260T | p.R1754X |  |
| ***ABCA7*** | 19:1051178 | [rs200408449](http://grch37.ensembl.org/Homo_sapiens/ZMenu/Variation?v=rs200408449) | c.G2709A | p.W903X |  |
| ***ABCA7*** | 19:1047631 | NA | c.G2247A | p.W749X |  |
| ***ABCA7*** | 19:1056244 | rs113809142 | c.4416+2T>G | NA | associated with LOAD Islandic population (2), reported in the Belgian population but not associated to LOAD (1) |

**Table S10**. **Nonsense mutations in *ABCA7* detected in our cohort and eliminated by the QC filter**. Position is in hg19/GRCh37.

| **GENE** | **TRANSCRIPT** | **POSITION** | **MA** | **Aa**  **change** | **Rs ID** | **ExAC**  **MAF(%)** | **POLYPHEN** | **GERP***  **score** | **Grantham****  **score** | **P-value** | **OR (95% CI)** | **Disease** | **Ref.** |
| --- | --- | --- | --- | --- | --- | --- | --- | --- | --- | --- | --- | --- | --- |
| ***IL23R*** | NM_144701.2 | [1:67635211](http://genome.ucsc.edu/cgi-bin/hgTracks?db=hg19&org=human&position=chr1%3A67634711-67635711&hgt.customText=http://evs.gs.washington.edu/evs_bigwig/chr1.multiTracks.txt) | G>A | p.R86Q | [rs76575803](http://www.ncbi.nlm.nih.gov/projects/SNP/snp_ref.cgi?searchType=adhoc_search&type=rs&rs=rs76575803) | 0.2776 | Benign | -2.52 | 43 | 0.010 | 0.40 (0.000-1.031) | CD | (3) |
| ***IL23R*** | NM_144701.2 | [1:67648596](http://genome.ucsc.edu/cgi-bin/hgTracks?db=hg19&org=human&position=chr1%3A67648096-67649096&hgt.customText=http://evs.gs.washington.edu/evs_bigwig/chr1.multiTracks.txt) | G>A | p.G149R | [rs76418789](http://www.ncbi.nlm.nih.gov/projects/SNP/snp_ref.cgi?searchType=adhoc_search&type=rs&rs=rs76418789) | 0.7084 | Probably-damaging | 5.24 | 125 | 1.46x10^-3^ | 0.335 (0.000–0.843) | CD, UL | (3)(4) |
| ***IL23R*** | NM_144701.2 | [1:67705900](http://genome.ucsc.edu/cgi-bin/hgTracks?db=hg19&org=human&position=chr1%3A67705400-67706400&hgt.customText=http://evs.gs.washington.edu/evs_bigwig/chr1.multiTracks.txt) | G>A | p.V362I | [rs41313262](http://exac.broadinstitute.org/variant/1-67705900-G-A) | 1.168 | Benign | -6.22 | 29 | 2.89x10^-4^ | 0.567 (0.000–0.821) | CD, UL | (3)(4) |
| ***IL23R*** | NM_144701.2 | [1:67705958](http://genome.ucsc.edu/cgi-bin/hgTracks?db=hg19&org=human&position=chr1%3A67705458-67706458&hgt.customText=http://evs.gs.washington.edu/evs_bigwig/chr1.multiTracks.txt) | G>A | p.R381Q | [rs11209026](http://www.ncbi.nlm.nih.gov/projects/SNP/snp_ref.cgi?searchType=adhoc_search&type=rs&rs=rs11209026) | 4.221 | Probably-damaging | 5.19 | 43 | <1.00x10^-6^ | 0.363 (0.000–0.452) | CD, UL | (3) |
| ***IFIH1*** | NM_022168.3 | [2:163124637](http://genome.ucsc.edu/cgi-bin/hgTracks?db=hg19&org=human&position=chr2%3A163124137-163125137&hgt.customText=http://evs.gs.washington.edu/evs_bigwig/chr2.multiTracks.txt) | T>C | I923V | rs35667974 | 1.156 | Probably-damaging | 5.13 | 29 | 2.1x10^-16^ | 0.51 (0.43 – 0.61) | T1D | (5) |
| ***IFIH1*** | NM_022168.3 | [2:163134090](http://genome.ucsc.edu/cgi-bin/hgTracks?db=hg19&org=human&position=chr2%3A163133590-163134590&hgt.customText=http://evs.gs.washington.edu/evs_bigwig/chr2.multiTracks.txt) | C>A | E627X | rs35744605 | 0.0008288 | NA | 2.87 | NA | 1.3x10^-3^ | 0.69 (0.52 – 0.91) | T1D | (5) |
| ***G6PC2*** | NM_021176.2 | [2:169764176](http://genome.ucsc.edu/cgi-bin/hgTracks?db=hg19&org=human&position=chr2%3A169763676-169764676&hgt.customText=http://evs.gs.washington.edu/evs_bigwig/chr2.multiTracks.txt) | G>C | p.V219L | rs492594 | 48.65 | Benign | 3.06 | 32 | NA | NA | T2D | (6) |
| ***TMEM106B*** | NM_001134232.1 | [7:12269417](http://genome.ucsc.edu/cgi-bin/hgTracks?db=hg19&org=human&position=chr7%3A12268917-12269917&hgt.customText=http://evs.gs.washington.edu/evs_bigwig/chr7.multiTracks.txt) | C>G | p.T185S | [rs3173615](http://www.ncbi.nlm.nih.gov/projects/SNP/snp_ref.cgi?searchType=adhoc_search&type=rs&rs=rs3173615) | 46.16 | Benign | 5.36 | 58 | NA | NA | FTLD | (7) |
| ***ABCA7*** | NM_019112 | 19:1043103 | G>A | p.G215S | rs72973581 | 4.31 | Benign | -5.86 | 56 | 6x10^-4^ | 0.57 (0.41-0.80) |  |  |

**Table S11**. **Protective variants reported at the GWAS loci**. Position is in hg19/GRCh37. MAF, minor allele frequency; Aa, amino acid; OR, odds ratio; Ref., reference; CD, Crohn’s disease; UL, ulcerative colitis; FTLD, frontotemporal lobar degeneration. .*GERP score >5 indicates high conservation among different species.**Grantham score < 50 indicates an amino acid substitution that does not importantly alter the protein sequence.

| **GENE** | **TRANSCRIPT** | **POSITION** | **MA** | **Aa**  **change** | **rs ID** | **ExAC**  **MAF(%)** | **POLYPHEN** | **GERP***  **score** | **Grantham****  **score** | **P-value** | **OR** | **EFFECT** | **Ref.** |
| --- | --- | --- | --- | --- | --- | --- | --- | --- | --- | --- | --- | --- | --- |
| ***ABCA1*** | NM_005502.3 | 9:107599376 | A>G | V399A | [rs9282543](http://exac.broadinstitute.org/variant/9-107599376-A-G) | 0.3520 | Benign |  |  | 0.6273 | 0.38 | Potential protective factor for AD | (8) |
|  |  |  |  |  |  |  |  | 5.7 | 64 |  |  |  |  |
| ***ABCA1*** | NM_005502.3 | 9:107589255 | C>T | V771M | [rs2066718](http://exac.broadinstitute.org/variant/9-107589255-C-T) | 5.11 | Benign | 4.55 | 21 | 0.1495 | 0.43 | Potential protective factor for AD | (8) |
| ***ABCA1*** | NM_005502.3 | 9:104819652 | G>A | P1059S | [rs371168450](http://www.ensembl.org/Homo_sapiens/Variation/Summary?db=core;g=ENSG00000165029;r=9:104781002-104928237;t=ENST00000374736;v=rs371168450;vf=53674331;source=dbSNP) | NA | Probably damaging | NA | NA | 0.04705 | 0.49 | Potential protective factor for AD | (8) |
| ***ABCA1*** | NM_005502.3 | [9:107579632](http://genome.ucsc.edu/cgi-bin/hgTracks?db=hg19&org=human&position=chr9%3A107579132-107580132&hgt.customText=http://evs.gs.washington.edu/evs_bigwig/chr9.multiTracks.txt) | C>G | E1172D | [rs33918808](http://www.ncbi.nlm.nih.gov/projects/SNP/snp_ref.cgi?searchType=adhoc_search&type=rs&rs=rs33918808) | 3.82 | Benign | 4.68 | 45 | 1 | 0.96 | Potential protective factor for AD | (8) |

**Table S12**. **Potential protective variants for AD reported in *ABCA1*** (8). Position is in hg19/GRCh37. MAF, minor allele frequency; Aa, amino acid; OR, odds ratio; Ref., reference; AD, Alzheimer’s disease.

*GERP score >5 indicates high conservation among different species

**Grantham score < 50 indicates an amino acid substitution that does not importantly alter the protein sequence

| *ABCA7* | 197 | L-R-S-L-V-E-L-R-A-L-L-Q-R-P-R-G-T-S-**G**-P-L-E-L-L-S-E-A-L-C-S-V-R-G-P-S-S-T |
| --- | --- | --- |
| *ABCA2* | 241 | T-P-G-S-G-E-L-G-R-I-L-T-V-P-E-S-Q-K-**G**-A-L-Q-G-Y-R-D-A-V-C-S-G-Q-A |
| *ABCA12* | 701 | R-S-V-P-L-T-Q-A-M-Y-R-S-N-R-M-N-T-P-Q-**G**-S-F-S-T-I-S-Q-A-L-C |
| *ABCA13* | 3217 | L-L-E-T-L-D-F-Q-Q-V-S-Q-N-V-Q-A-R-S-S-A-F-**G**-S-F-Q-F-V-M-K-M-V-C—K-D-Q-A-S-F |
| *ABCA1* | 274 | M-R-S-W-S-D-M-R-Q-E-V-M-F-L-T-N-V-N-S-**S**-S-S-S-T-Q-I-Y-Q-A-V-S-R |

| *Homo Sapiens* | 197 | L-R-S-L-V-E-L-R-A-L-L-Q-R-P-R-G-T-S-**G**-P-L-E-L-L-S-E-A-L-C-S-V-R-G-P-S-S-T |
| --- | --- | --- |
| *Gorilla* | 197 | L-R-S-L-V-E-L-Q-A-L-L-R-R-P-R-G-T-S-**G**-P-L-E-L-L-S-E-A-L-C-S-A-R-G-P-S-S-T |
| *Macaca mulatta* | 195 | L-P-S-L-G-E-L-W-A-L-L-Q-R-P-H-R-P-G-**G**-P-L-E-A-V-A-E-A-L-C-S-A-R-G-P-S-K-P |
| *Bos Taurus* | 197 | L-P-S-L-V-E-L-Q-A-L-L-H-R-P-R-G-T-G-**G**-P-L-E-L-L-S-E-A-L-C-S-A-R-G-P-S-S-T |
| *Rattus norvegicus* | 193 | L-P-S-L-V-E-L-R-A-L-L-R-R-P-Q-G-P-G-**G**-P-L-E-A-V-S-E-A-L-C-G-A-R-G-P-G-I-P |
| *Mus musculus* | 194 | L-P-S-L-M-E-L-R-A-L-L-R-R-P-R-G-S-A-**G**-S-L-E-L-V-S-E-A-L-C-S-T-K-G-P-S-S-P |
| *Canis lupus* | 194 | L-P-S-L-V-E-L-R-A-L-L-R-R-P-Q-G-T-R-**S**-P-L-Q-L-V-S-E-A-F-C-S-A-K-G-P-S-S-P |
| *Pteropus vampyrus* | 191 | L-P-S-L-A-E-L-Q-A-L-L-P-R-L-R-E-T-D-**S**-T-L-A-V-V-S-E-A-L-C-S-A-K-G-P-S-V-P-G-G-P-S |
| *Cavia porcellus* | 272 | L-P-S-L-A-E-L-Q-A-L-L-Q-R-P-W-G-T-S-**S**-S-L-E-L-V-S-E-A-L-C-S-A-K-G-P-S-S-P-G-G |

**Figure S2**. **Conservation of ABCA7 p.G215 in homologous proteins**

**Figure S1**. **Conservation of ABCA7 p.G215 in different species**

**Bioinformatic**

Each of the samples in our dataset consisted of paired-end 100 base pair reads. We used the Burrows-Wheeler Aligner (BWA)(9) to map the reads to build of the human genome (hg19/GRCh37). Following read mapping, we used SAMtools (10), Picard (<http://picard.sourceforge.net>), and the Genome Analysis Toolkit (GATK) (11)(12) to refine the resulting alignments by removing duplicates, performing realignment around InDels, and recalibrating base quality scores. We then used the GATK’s UnifiedGenotyper to identify sequence variants, and subsequently filtered the variants and recalibrated variant quality scores (11). Our final dataset consisted of variant call format (VCF) files containing variants that passed all filters. Since our dataset consisted of a mix of exomes captured using different kits, and whole genome sequences, we employed a highly conservative approach to variant selection to increase our confidence that analyzed variants are true positives. We limited our dataset of variants to only those genomic regions we expected to have been sequenced in each of the exomes (based on capture probes used for exome library preparation) and whole genomes. Next, we compiled a list of all the variants present in at least a single sample. We examined each of the variants from the list of total variants in each sample, whether or not the variant was called by the GATK, and reassigned the genotype for that variant according to the following criteria. (1) If the variant was called by the GATK and passed all filters, we used the GATK genotype. (2) If no variant was called at the genomic position in question, we returned to the raw VCF file and if there were reads containing the variant, but the variant was not called because of failing filters or because only a small number of reads contain the variant, we set the genotype to missing for the sample. (3) Finally, if all the reads at this position for the sample indicated reference alleles, we set the genotype to homozygous reference. Resulting sequence files were converted to Plink format (13) using VCFTools (14). Lastly, we removed all variants not in our pre-defined list of candidate genes (*ABCA7* [NM_019112]; *CD2AP* [NM_012120]; *MS4A6A* [NM_152851]; *CR1* [NM_000573]; *BIN1* [NM_139343]; *PICALM* [NM_001206946]; *EPHA1* [NM_005232]; *CLU* [NM_001831]; *CD33* [NM_001772]. Remaining variants were annotated using ANNOVAR (15). Each variant was annotated with gene information (gene name, transcript ID, and transcript and protein positions of the variant), genomic location (exon, intron, UTR, intergenic, etc.), one or more variant classes (5’-UTR, 3’-UTR, intergenic, intronic, splice site, nonsynonymous, stop-gain, stop-loss, or synonymous), the 1000 Genomes minor allele frequency (16), dbSNP identifier(17), and PolyPhen-2 (18).

**References**

1. Cuyvers E, De Roeck A, Van den Bossche T, Van Cauwenberghe C, Bettens K, Vermeulen S, et al. Mutations in ABCA7 in a Belgian cohort of Alzheimer’s disease patients: a targeted resequencing study. Lancet Neurol. 2015 Aug;14(8):814–22.

2. Steinberg S, Stefansson H, Jonsson T, Johannsdottir H, Ingason A, Helgason H, et al. Loss-of-function variants in ABCA7 confer risk of Alzheimer’s disease. Nat Genet. 2015 Mar 25;

3. Momozawa Y, Mni M, Nakamura K, Coppieters W, Almer S, Amininejad L, et al. Resequencing of positional candidates identifies low frequency IL23R coding variants protecting against inflammatory bowel disease. Nat Genet. 2011 Jan;43(1):43–7.

4. Rivas MA, Beaudoin M, Gardet A, Stevens C, Sharma Y, Zhang CK, et al. Deep resequencing of GWAS loci identifies independent rare variants associated with inflammatory bowel disease. Nat Genet. 2011 Nov;43(11):1066–73.

5. Nejentsev S, Walker N, Riches D, Egholm M, Todd JA. Rare variants of IFIH1, a gene implicated in antiviral responses, protect against type 1 diabetes. Science. 2009 Apr 17;324(5925):387–9.

6. Mahajan A, Sim X, Ng HJ, Manning A, Rivas MA, Highland HM, et al. Identification and functional characterization of G6PC2 coding variants influencing glycemic traits define an effector transcript at the G6PC2-ABCB11 locus. PLoS Genet. 2015 Jan;11(1):e1004876.

7. Nicholson AM, Finch NA, Wojtas A, Baker MC, Perkerson RB, Castanedes-Casey M, et al. TMEM106B p.T185S regulates TMEM106B protein levels: implications for frontotemporal dementia. J Neurochem. 2013 Sep;126(6):781–91.

8. Lupton MK, Proitsi P, Lin K, Hamilton G, Daniilidou M, Tsolaki M, et al. The role of ABCA1 gene sequence variants on risk of Alzheimer’s disease. J Alzheimers Dis JAD. 2014;38(4):897–906.

9. Li H, Durbin R. Fast and accurate short read alignment with Burrows-Wheeler transform. Bioinforma Oxf Engl. 2009 Jul 15;25(14):1754–60.

10. Li H, Handsaker B, Wysoker A, Fennell T, Ruan J, Homer N, et al. The Sequence Alignment/Map format and SAMtools. Bioinforma Oxf Engl. 2009 Aug 15;25(16):2078–9.

11. DePristo MA, Banks E, Poplin R, Garimella KV, Maguire JR, Hartl C, et al. A framework for variation discovery and genotyping using next-generation DNA sequencing data. Nat Genet. 2011 May;43(5):491–8.

12. McKenna A, Hanna M, Banks E, Sivachenko A, Cibulskis K, Kernytsky A, et al. The Genome Analysis Toolkit: a MapReduce framework for analyzing next-generation DNA sequencing data. Genome Res. 2010 Sep;20(9):1297–303.

13. Purcell S, Neale B, Todd-Brown K, Thomas L, Ferreira MAR, Bender D, et al. PLINK: a tool set for whole-genome association and population-based linkage analyses. Am J Hum Genet. 2007 Sep;81(3):559–75.

14. Danecek P, Auton A, Abecasis G, Albers CA, Banks E, DePristo MA, et al. The variant call format and VCFtools. Bioinforma Oxf Engl. 2011 Aug 1;27(15):2156–8.

15. Wang K, Li M, Hakonarson H. ANNOVAR: functional annotation of genetic variants from high-throughput sequencing data. Nucleic Acids Res. 2010 Sep;38(16):e164.

16. 1000 Genomes Project Consortium, Abecasis GR, Auton A, Brooks LD, DePristo MA, Durbin RM, et al. An integrated map of genetic variation from 1,092 human genomes. Nature. 2012 Nov 1;491(7422):56–65.

17. Sherry ST, Ward MH, Kholodov M, Baker J, Phan L, Smigielski EM, et al. dbSNP: the NCBI database of genetic variation. Nucleic Acids Res. 2001 Jan 1;29(1):308–11.

18. Adzhubei IA, Schmidt S, Peshkin L, Ramensky VE, Gerasimova A, Bork P, et al. A method and server for predicting damaging missense mutations. Nat Methods. 2010 Apr;7(4):248–9.
